# Identification and Functional Characterization of a Microtubule-Associated Protein, GhCLASP2, From Upland Cotton (*Gossypium hirsutum* L.)

**DOI:** 10.3389/fpls.2018.00882

**Published:** 2018-06-27

**Authors:** Shou-Hong Zhu, Fei Xue, Yan-Jun Li, Feng Liu, Xin-Yu Zhang, Lan-Jie Zhao, Yu-Qiang Sun, Qian-Hao Zhu, Jie Sun

**Affiliations:** ^1^The Key Laboratory of Oasis Eco-Agriculture, College of Agriculture, Shihezi University, Shihezi, China; ^2^State Key Laboratory of Cotton Biology, Institute of Cotton Research, Chinese Academy of Agricultural Sciences, Anyang, China; ^3^Zhejiang Province Key Laboratory of Plant Secondary Metabolism and Regulation, College of Life Sciences, Zhejiang Sci-Tech University, Hangzhou, China; ^4^CSIRO Agriculture and Food, Canberra, ACT, Australia

**Keywords:** *GhCLASP2*, microtubule, trichome, cotton fiber, fiber strength, *G. hirsutum*

## Abstract

Cytoplasmic linker-associated proteins (CLASPs) are microtubule-associated proteins (MAPs) involved in regulation of dynamics of microtubules (MTs) that play an important role in plant growth and development. In this study, we identified cotton *CLASP* genes and investigated the function of *GhCLASP2*. *GhCLASP2* was mainly expressed in stem and developing fibers, especially in fibers of the secondary cell wall deposition stage. Ectopic expression of *GhCLASP2* in *Arabidopsis* increased the branching number of leaf trichomes and rescued the defective phenotypes of *clasp-1*. In cotton, overexpression of *GhCLASP2* increased fiber strength, probably related to enhanced expression levels of tubulin, cellulose synthase, and expansin genes. Suppression of *GhCLASP2* caused shorter internodes and semi-dwarfism, abnormal flower stigma, aborted anthers without pollen grains, and sterility. These changed phenotypes were similar to those observed in the *Arabidopsis clasp-1* mutant. GhCLASP2 was co-localized with MTs according to transient experiment. These results suggest that *GhCLASP2* functions similarly as *AtCLASP*, acting as a MAP and controlling cotton growth and development by regulating MTs.

## Introduction

As a component of the cytoskeleton in eukaryotic cells, microtubules (MTs) play a vital role in cell life, such as cell morphogenesis and maintenance, intracellular transport, cell migration, cell division, and signal transduction. Their functions are accomplished with a variety of MT-associated proteins (MAPs). Interaction between MTs and MAPs regulates the aggregation and depolymerization of MTs, the orientation changes of MT arrays, and the dynamic activities of MTs through spatial organization of MT arrays. MAPs control cell division and expansion, two fundamental biological processes crucial for growth and development of all eukaryotes (Gardiner, 2013; Hamada, 2014).

Cytoplasmic linker-associated proteins (CLASPs) are members of the ORBIT/MAST/CLASP family of MAPs, which bind to MTs and associate with the cytoplasmic linker proteins (CLIPs), such as CLIP-170 and EB1 (Akhmanova et al., 2001). CLASP was observed anywhere along the entire length of MTs, but mostly concentrated at the plus end of MTs (Kirik et al., 2007; Muller et al., 2009), they thus belong to a diverse group of molecules named MT plus-end-tracking proteins (+TIPs) that specifically accumulate at the plus ends of growing MTs (Akhmanova and Hoogenraad, 2005; Ruan and Wasteneys, 2014). CLASPs have been shown to play various functions in animals and yeast, including stabilizing the MT plus ends (Mimori-Kiyosue et al., 2005), promoting MT rescue by recruiting tubulin dimers (Al-Bassam et al., 2010), attaching MT plus ends to the cell cortex (Lansbergen et al., 2006), regulating persistent motility of MT bundling (Stramer et al., 2010), and nucleating noncentrosomal MTs from *trans*-Golgi bodies (Efimov et al., 2007). It has been proposed that CLASPs mediate interaction between the plus ends of cytoplasmic MTs radiating from the centrosome to specific cortical sites at the plasma membrane, where they act as cortical anchors for the plus ends of MTs (Mimori-Kiyosue et al., 2005; Lansbergen et al., 2006). CLASPs also stabilize cortical MT at the cell edge through inhibition of MT depolymerization (Ambrose and Wasteneys, 2008; Ambrose et al., 2011). Kinesin-13s, members of the kinesin superfamily of motor proteins, make MTs unstable and increase the chance of catastrophe as a result of MT depolymerization. In contrast, CLASPs promote MT rescue, i.e., preventing MTs from shrinkage or catastrophe and regulate MT’s polymerization status and the stability of MT dynamics to maintain the activities of cell life (Al-Bassam et al., 2010; Al-Bassam and Chang, 2011; De Forges et al., 2016).

The *Arabidopsis* genome contains only a single copy of *CLASP* and no *CLIP* (Ambrose et al., 2007). As in animals and yeast (Kirik et al., 2007; Ambrose and Wasteneys, 2008; Ambrose et al., 2011), AtCLASP is localized at the plus ends of growing MTs and is essential for the proper organization of spindle and phragmoplast (Hamada, 2014). The *Arabidopsis clasp-1* mutant exhibited dwarfism, no obvious apical dominance, lower seed setting percentage, shorter pods, reduced trichome branching, slower growth of hypocotyls and roots, reduced size of division and elongation zone in roots, and slower cell elongation rate (Ambrose et al., 2007, 2013). When treated with MT depolymerization drugs, the roots of *clasp-1* showed abnormal swelling at a lower concentration of drugs compared to that of wild-type, suggesting the mutant is more sensitive to MT depolymerization drugs than wild-type (Ambrose et al., 2007). These results indicated that CLASP is essential for proper cell division and expansion, and involved in establishment of plant cell morphology. The *clasp-1* mutant also showed excessive root branching, a phenomenon related to defective auxin pathways (Ambrose et al., 2007; Kirik et al., 2007). Further investigations demonstrated that *clasp-1* displayed auxin-related phenotypes and AtCLASP interacts with the retromer component sorting nexin 1 (SNX1) to regulate PIN2 auxin transporter trafficking and stability (Ambrose et al., 2013; Brandizzi and Wasteneys, 2013; Kakar et al., 2013; Zhang et al., 2013). The relationship between the transport of the plasma membrane substances and MTs found in these studies revealed an auxin signaling mechanism regulated by MT cytoskeleton (Brandizzi and Wasteneys, 2013; Kakar et al., 2013; Zhang et al., 2013; Ruan and Wasteneys, 2014; Brumbarova and Ivanov, 2016). Furthermore, AtCLASP interacts with AtSABRE to stabilize MTs and guides the orientation of plant cell division and cell polarity (Pietra et al., 2013; Zhang et al., 2013; Ruan and Wasteneys, 2014; Van Dop et al., 2015; Brumbarova and Ivanov, 2016; Qi and Greb, 2017).

Cotton is a globally important economic crop. Its fiber is a valuable natural material for the textile industry. Each cotton fiber is an extremely elongated single cell initiated and developed from the epidermal layer of ovule. Cotton fiber serves as an excellent biological model for investigation of mechanisms underlying the process of cell development, particularly cell elongation. Both elongation and synthesis of secondary cell walls of fiber cells are closely related to the arrangement of the periplasmic MTs (Dixit and Cyr, 2004a,b). The number of MTs increased significantly during thickening of secondary cell walls, and the orientation of MT arrays was consistent with the direction of the newly formed fibrous layer (Yatsu and Jacks, 1981). Cortical MTs provide spatial information necessary for the alignment of cellulose microfibrils that confine and regulate cell elongation. During elongation of cotton fiber cells, reorientation of microfibrils caused by orientation change of MT arrays increases accumulation of microfibrils in the fiber cell walls (Seagull, 1986, 1992). MTs are cytoskeletal structures for orbital transport of substances within cells, when MTs are destroyed; substance transportation within cells is inhibited. MTs thus are expected to play an important role in the development of cotton fibers, so are CLASPs because they are a type of MAPs involved in the formation and development of MTs. However, little is known about cotton *CLASP* genes, including their numbers in the cotton genomes and functions in cotton development, particularly in cotton fiber development.

In this study, we identified cotton *CLASP* genes and investigated the function of *GhCLASP2* using transgenic cottons with an elevated or knocked down level of *GhCLASP2*. Our results demonstrated a conserved function of *GhCLASP2* and *AtCLASP*, and revealed a potential role of *GhCLASP2* in enhancing fiber strength.

## Materials and Methods

### Plant Materials and Growth

Cotton (*Gossypium hirsutum* L.) cultivars of “Xinluzao33” and “YZ-1” were used in this study. Cotton plants were grown in the experimental field of Shihezi University (Shihezi, Xinjiang, China). Flower buds on the anthesis day were tagged and marked as 0 day post anthesis (DPA). Fibers of 3, 6, 9, 12, 15, 18, 21, 24, 27, and 30 DPA bolls were peeled off from the ovules and used in gene expression analysis. Seeds of Xinluzao33 were decoated and surface sterilized with 0.1% (w/v) HgCl_2_ solution for 10 min, and then washed three to four times with sterile water and grown on the 1/2 Murashige and Skoog medium (MS medium) under a 16 h light/8 h dark cycle at 28°C. Roots, stems, and leaves were collected from 2-week-old seedlings. The collected materials were immediately frozen in liquid nitrogen for RNA extraction.

The ecotype Columbia (Col-0) of *Arabidopsis thaliana* and the mutant *clasp-1* (Salk_120061) were grown in the growth chambers with a 16 h light/8 h dark cycle at 22°C for observation and transformation by floral dip (Clough and Bent, 1998). Phenotypic observation was performed at the same growth period of different transgenic lines. Thirty leaves collected from the same position (the eighth rosette leave) of each plant were used in counting the number of epidermal trichomes and data analysis. *Nicotiana benthamiana* plants were grown in the growth chambers with a 16 h light/8 h dark cycle at 22°C and used in observation of subcellular localization of GhCLASP2.

### Isolation of Total RNA, Semi-quantitative PCR, and qRT-PCR Analysis

Total RNA was extracted from cotton roots, stems, leaves, flowers, and fibers of different developmental stages. cDNA was synthesized by using an EASYspin Plus Plant RNA Kit (Aidlab) with gDNA Eraser (Takara). The expression levels of *GhCLASPs* were analyzed by semi-quantitative PCR (sqPCR) and quantitative real-time RT-PCR (qRT-PCR). The cotton *UBQ7* gene was used as a standard control in both sqPCR and qRT-PCR. The gene-specific primers used in the study were shown in Supplementary Table 1. sqPCR was performed using cDNA template and ExTaq DNA Polymerase (Takara) by 28 cycles at an annealing temperature of 58°C. The qRT-PCR reactions were conducted using a SYBR Green I Master mixture (Roche, Basel, Switzerland) according to the manufacturer’s protocol on a Light Cycler 480II system (Roche, Switzerland) under conditions of an initial denaturation at 95°C for 2 min followed by 40 cycles of denaturing at 95°C for 15 s, annealing at 58°C for 20 s, and extending at 72°C for 15 s. For each tissue, three biological replicates each with three technical replicates were analyzed. The relative expression levels of genes were performed using the 2^-ΔΔc_t_^ method (Livak and Schmittgen, 2001).

### Identification and Sequence Analysis of *GhCLASP2*

The protein sequence of *AtCLASP* (At2g20190) was used as a query to search against the annotated proteins of *G. arboreum* (A2, BGI, version 1.0), *G. raimondii* (D5, JGI, version 2.0), and *G. hirsutum* (AD1, NAU, version 1.1) to identify *CLASP* genes in these three cotton species. The blastp search was done in COTTONGEN^1^ (Yu et al., 2014). The full-length cDNA of *GhCLASP2* was amplified using primers (Supplementary Table 2) designed based on the sequence of its *G. raimondii* homolog *Gorai.001G235400.1*.

Multiple alignment of the deduced amino acid sequences of the identified CLASP genes was performed using the ClustalW2 at EMBL-EBI^2^. The phylogenetic relationship tree was constructed by the neighbor-joining (NJ) method implemented in the MEGA (version 6.0) software. Prediction of the CLASP domain, theoretical isoelectric point (pI), and molecular weight was done using the web-based protein database, Expert Protein Analysis System (ExPASy^3^).

### Generation and Identification of Transgenic *Arabidopsis* Plants

The *GhCLASP2*-overexpression vector was constructed using the PCR product amplified using primers of *GhCLASP2*-OE-F (with the *Kpn*I restriction site) and *GhCLASP2*-OE-R (with the *Bam*HI restriction site) (Supplementary Table 2). The amplified *Kpn*I-*GhCLASP2*-*Bam*HI product was recombined into the *Kpn*I and *Bam*HI restriction sites of the pCAMBIA2300-ubiquitin-ocs vector. The successful construction of the *GhCLASP2*-overexpression vector was confirmed by digestion with *Kpn*I and *Bam*HI and sequencing. *Agrobacterium* (strain GV3101) containing the *GhCLASP2*-overexpression vector was used to transform *Arabidopsis* wild-type Col-0 and its mutant *clasp-1* plants (Clough and Bent, 1998) to generate *Arabidopsis* lines overexpressing *GhCLASP2* and complementing the *clasp-1* mutation, respectively. Homozygous T_3_ transgenic *Arabidopsis* lines were identified by PCR and kanamycin (50 mg/l) selection.

Quantitative real-time RT-PCR was performed to evaluate the expression level of *GhCLASP2* in the transgenic *Arabidopsis* lines. Each 10 μl PCR reaction contained 5 μl 2× SYBR Green I Master Mix (Roche, Basel, Switzerland), 50 ng cDNA, and 0.4 μM of each primer specific for *GhCLASP2* (Supplementary Table 1). The *Arabidopsis Actin* gene was used as an internal control for expression normalization. qRT-PCR was performed in a Light Cycler 480II system (Roche, Switzerland) and the relative expression levels of genes were performed using the 2^-ΔΔc_t_^ method (Livak and Schmittgen, 2001).

### Subcellular Localization of GhCLASP2

To investigate the localization of the GhCLASP2 protein, the coding region of *GhCLASP2* was amplified using the primers of GFP-*GhCLASP2*-F (with the adaptor CACC) and GFP-*GhCLASP2*-R (Supplementary Table 2). The pGWB6-GFP-GhCLASP2 vector was generated by inserting the coding region of *GhCLASP2* into the pGWB6 vector using the Gateway technology. The BP reaction was catalyzed by the BP Clonase enzyme mix and the LR reaction was catalyzed by the LR Clonase enzyme (Invitrogen, United States) (Miki and Shimamoto, 2004; Nakagawa et al., 2007). The pGWB6-GFP-GhCLASP2 plasmid was then transformed into *Agrobacterium* (strain GV3101). Three-week-old tobacco (*N. benthamiana*) leaves were infiltrated with *Agrobacteria* (Kong et al., 2015). Subcellular localization was examined with a confocal microscope (Leica TCS SP5) at 2–4 d (days) after infiltration.

To further verify GhCLASP2 acting as a MAP and co-localized with MTs, the mCherry-TUB6 with a mCherry tag and the pGWB6-GFP-GhCLASP2 with a GFP tag were separately transformed into *Agrobacterium* (strain GV3101) and used in infiltration. Tobacco leaves were infiltrated as described above (the volume ratio of pGWB6-GFP-GhCLASP2 and mCherry-TUB6 *Agrobacteria* was 1:1, OD = 0.5) and the infiltrated leaves were investigated under a confocal microscope at 2–4 d after infiltration (Kong et al., 2015).

### Generation and Identification of Transgenic Cotton Plants

The surface sterilized seeds of “YZ-1” were cultured on the ½ MS medium at 28°C for 5–6 d in the dark. Hypocotyls of seedlings were cut into sections of 6–8 mm and used as explants for *Agrobacterium*-mediated transformation. The *GhCLASP2*-RNAi vector was constructed using a PCR fragment amplified by *GhCLASP2* specific primers *GhCLASP2*-RNAi-F (with the adaptor CACC) and *GhCLASP2*-RNAi-R (Supplementary Table 2). The amplified *GhCLASP2* product was recombined into the PANDA35HK vector using the Gateway technology as mentioned above (Miki and Shimamoto, 2004; Nakagawa et al., 2007). *Agrobacterium* (strain LB4404) containing the *GhCLASP2*-overexpression vector or the *GhCLASP2*-RNAi vector was used to infect cotton hypocotyls. Calli induction and plant regeneration were performed based on a published protocol (Jin et al., 2006). Transgenic cotton plants were screened by PCR and antibiotic kanamycin (50 mg/l). The expression level of *GhCLASP2* in the transgenic plants was analyzed using sqRT-PCR and qRT-PCR. *GhUBQ7* was used as a standard control in both sqRT-PCR and qRT-PCR.

### Quality Measurement of Cotton Fiber

Fiber samples (10 g each) were chosen randomly from each T_3_ transgenic line overexpressing *GhCLASP2* and wild-type plants after ginning. Fiber quality analysis was done using the High Volume Instrument in the Center of Cotton Fiber Quality Inspection and Testing, Chinese Ministry of Agriculture (Anyang, Henan, China).

## Results

### Identification of Cotton *CLASP* Genes

**FIGURE 1:**
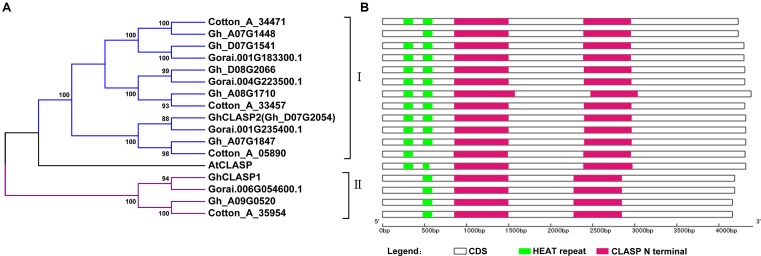
Phylogenetic relationship and protein domain organization of CLASP family member from three cotton species and *Arabidopsis*. **(A)** Phylogenetic relationship of CLASP proteins from three cotton species and *Arabidopsis*. The tree was generated using the NJ approach based on the amino acid sequences of CLASPs. The numbers next to each node indicate bootstrap values from 1000 replicates. Cotton_A_35954, Cotton_A_34471, Cotton_A_33457, and Cotton_A_05890 are from *G*. *arboreum*; Gorai.001G183300.1, Gorai.001G235400.1, Gorai.006G054600.1, and Gorai.004G223500.1 are from *G*. *raimondii*; Gh_A07G1448, Gh_D07G1541, Gh_A09G0520, Gh_D08G2066, Gh_A08G1710, and Gh_A07G1847 are from *G*. *hirsutum* acc.TM-1; GhCLASP1 and GhCLASP2 (Gh_D07G2054) are from *G*. *hirsutum* acc.Xinluzao33; and AtCLASP is from *Arabidopsis*. **(B)** Protein domain organization of the CLASP family members in cotton. The CLASP N terminal domain and HEAT repeats are represented by red and green bars, respectively.

We started cotton CLASP gene-related work when only the *G*. *raimondii* genome sequence was available. We searched the annotated proteins of *G*. *raimondii* (D5) (Paterson et al., 2012) using the protein sequence of AtCLASP (At2g20190), and identified four putative cotton *CLASP* genes (*Gorai.001G183300*, *Gorai.001G235400*, *Gorai.004G223500*, and *Gorai.006G054600*). Two *CLASP* genes, *GhCLASP1* (GenBank ID: KP742966) (Zhu et al., 2015) and *GhCLASP2* (GenBank ID: MF991943), were then cloned in *G*. *hirsutum* cv. Xinluzao33 using primers based on the *G*. *raimondii CLASP* genes (Supplementary Table 2). *GhCLASP1* and *GhCLASP2* were similar to *Gorai.006G054600* and *Gorai.001G235400*, respectively. We further searched the annotated proteins of *G*. *arboreum* (A2) and *G*. *hirsutum* (AD1) when their genome sequences were published (Li et al., 2014; Zhang et al., 2015), and found four and seven homologous *CLASPs* in *G*. *arboreum* and *G*. *hirsutum*, respectively. Based on phylogenetic analysis, these cotton *CLASP* genes formed two distinct groups: group I and group II (**Figure 1A**). Each *G*. *arboreum* and *G*. *raimondii CLASP*, except *Gorai.006G054600* of *G*. *raimondii* from group II, had a homolog identified in *G*. *hirsutum* cv. TM-1 (Zhang et al., 2015; **Figure 1A**). Because *GhCLASP1* we cloned from another *G*. *hirsutum* cv. Xinluzao33 is a homolog of *Gorai.006G054600* (Zhu et al., 2015), we reasoned that TM-1 should also contain *GhCLASP1*. To confirm this, we searched the annotated proteins of the US version of the TM-1 genome (unpublished data^4^) using *GhCLASP1* as a query and found its corresponding gene, *Gohir.1Z037600*. The chromosome location of *Gohir.1Z037600* has not been determined (located on scaffold_255), but it likely belongs to chromosome D09 based on the location (chromosome A09) of its homoeolog *Gh_A09G0520*. We also searched another version of the TM-1 genome annotation (Li et al., 2015) and found only six *CLASP* genes with *CotAD_68468* and *CotAD_04861* corresponding to *GhCLASP1* and *GhCLASP2*, respectively (Zhu et al., 2017). *Gohir.1Z037600* and *GhCLASP1* had identical length and 11 single-nucleotide polymorphisms (SNPs; three synonymous and eight non-synonymous ones). Compared to *GhCLASP1*, *CotAD_68468* was 123-bp longer probably due to sequence misassembly and/or inclusion of introns with non-canonical splicing signals (Supplementary Figure 1). These results indicate that the assembly and/or annotation of different version of the TM-1 genome sequences were inconsistent, but *G*. *hirsutum* has retained all *CLASPs* derived from its ancestral diploid species and the *CLASP* genes are well conserved during cotton evolution.

Based on our previous results, the expression levels of the three *G*. *hirsutum CLASP* genes, including *GhCLASP2* or *CotAD_04861*, were gradually increased in 6–24 DPA fibers (Zhu et al., 2017). Inspired by this observation, in this study, we aimed to investigate the functionality of *GhCLASP2* and to know whether *GhCLASP2* plays a role in fiber development. *GhCLASP2* has a 4320-bp long coding sequence, encodes 1439 amino acids with a molecular mass of 158.95 kD, and a pI of 6.79 (Supplementary Table 3 and **Figure 1B**). GhCLASP2 and AtCLASP have a protein sequence identity of 75.16%.

The corresponding genes of *GhCLASP2* cloned from Xinluzao33 in the three versions of the TM-1 genome are *CotAD_04861* (Li et al., 2015), *Gh_D07G2054* (Zhang et al., 2015), and *Gohir.D07G208500* (unpublished data), which were annotated quite differently with a length of 3795, 4395, and 4320 bp (Supplementary Table 3), respectively. *Gohir.D07G208500* and *GhCLASP2* had identical length and four SNPs (two synonymous and two non-synonymous ones). Compared to *GhCLASP2*, *Gh_D07G2054* was 75-bp longer, and *CotAD_04861* was 525-bp shorter. Based on sequence alignment, it seems that the longer *Gh_D07G2054* was due to inclusion of a 75-bp intron although the exact reason for the shorter *CotAD_04861* was unclear. In addition, there were more “SNPs” between *GhCLASP2* and *Gh_D07G2054* and *CotAD_04861* (Supplementary Figures 2, 3). Based on these results, we believe that the sequence of *Gohir.D07G208500* was properly annotated while those of *Gh_D07G2054* and *CotAD_04861* were mis-annotated.

Cytoplasmic linker-associated proteins have two conserved domains: the HEAT repeat domain and the CLASP N terminal domain. The HEAT repeat domains have a helical structure, regulate interaction between proteins, and are involved in intracellular transport (Andrade and Bork, 1995; Groves et al., 1999). The CLASP N terminal domain is highly conserved, and contains MT-binding sites regulating the stability of MT dynamic (Grallert et al., 2006; Pereira et al., 2006). GhCLASPs showed high sequence identity among each other, particularly in the CLASP N terminal domain (Supplementary Figure 4). Like AtCLASP, most GhCLASPs contained two HEAT repeats and two CLASP N terminal domains (**Figure 1B**). However, all group II homoeologs contained only one HEAT repeat (Zhu et al., 2015) (**Figure 1B**). Two group I GhCLASPs, Gh_A07G1448 and Cotton_A_05890, also contained a single HEAT domain, and have lost the first and second HEAT domain due to non-synonymous nucleotide change and short sequence deletion, respectively (**Figure 1B**).

### *GhCLASP2* Is Preferentially Expressed in Cotton Fibers at the Secondary Wall Thickening Stage

**FIGURE 2:**
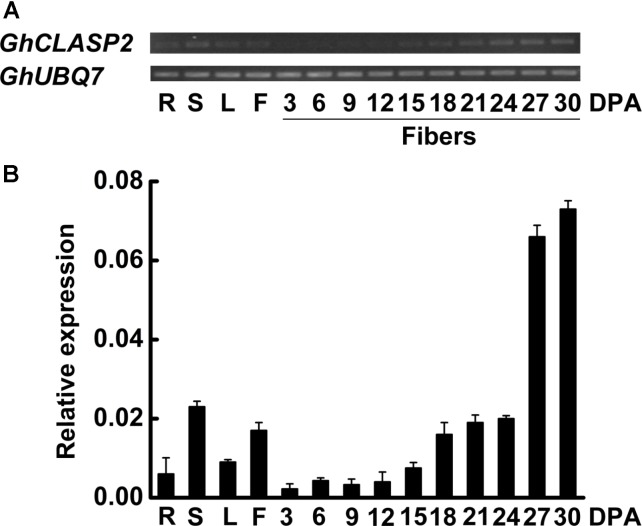
Expression patterns of *GhCLASP2*. **(A)** Expression level of *GhCLASP2* in various cotton tissues measured by sqPCR. **(B)** Expression level of *GhCLASP2* in various cotton tissues measured by qRT-PCR. R, root; S, stem; L, leaf; F, flower (0 DPA), and fibers of 3, 6, 9, 12, 15, 18, 21, 24, 27, and 30 DPA. Data are means ± SD. Error bars represent the standard deviation of triplicate experiments, and *GhUBQ7* was used as an internal control.

Each cotton fiber is a single celled seed trichome or hair. Cotton fibers have been used as a model for studying cell differentiation and development. The development of cotton fiber generally consists of four distinct but overlapping stages: fiber initiation, fiber cell elongation, biosynthesis of secondary cell wall cellulose, and maturation (Lee et al., 2007). Here, we first analyzed the expression patterns of *GhCLASP2* in vegetative, reproductive tissues, and more fiber samples to confirm our previous observation (Zhu et al., 2017). The expression level of *GhCLASP2* was much higher in stem and flower than in root and leaf. In developing fibers, the expression level of *GhCLASP2* increased gradually from 3 to 30 DPA, and a significantly increased expression was observed from 24 to 27 DPA, coincident with the transition from fiber elongation to secondary cell wall thickening (**Figure 2**). The expression patterns suggested a role of *GhCLASP2* in fiber development, especially in fiber secondary cell wall biosynthesis.

We also analyzed the expression patterns of other seven *GhCLASP2* homologs in different cotton organs (Supplementary Figure 5). The expression levels of the four pairs of homoeologs were quite different. For three homoeologous pairs (*Gh_A07G1448/Gh_D07G1541*, *Gh_A08G171/Gh_D08G2066*, and *Gh_A07G1847/GhCLASP2*), their At and Dt sub-genome homoeologs had a similar expression pattern in the tissues analyzed. The highest expression levels of *Gh_A07G1448* and *Gh_D07G1541* were observed in stem. Both pairs of *Gh_A08G1710*/*Gh_D08G2066* and *Gh_A07G1847/GhCLASP2* were predominantly expressed in fibers. But the expression patterns of *Gh_A09G0520* and its homoeolog *GhCLASP1* were different in the tissues analyzed (Supplementary Figure 5). *Gh_A09G0520* was highly expressed in flowers, whereas *GhCLASP1* was preferentially expressed in stem and 27 DPA fibers (Supplementary Figure 5). Different expression patterns suggest that these *CLASPs* might have different functions in cotton.

### The Ectopic Expression of *GhCLASP2* in *Arabidopsis* Increases the Branch Number of Leaf Trichomes and Rescues the Defective Phenotypes of the Mutant *clasp-1*

**FIGURE 3:**
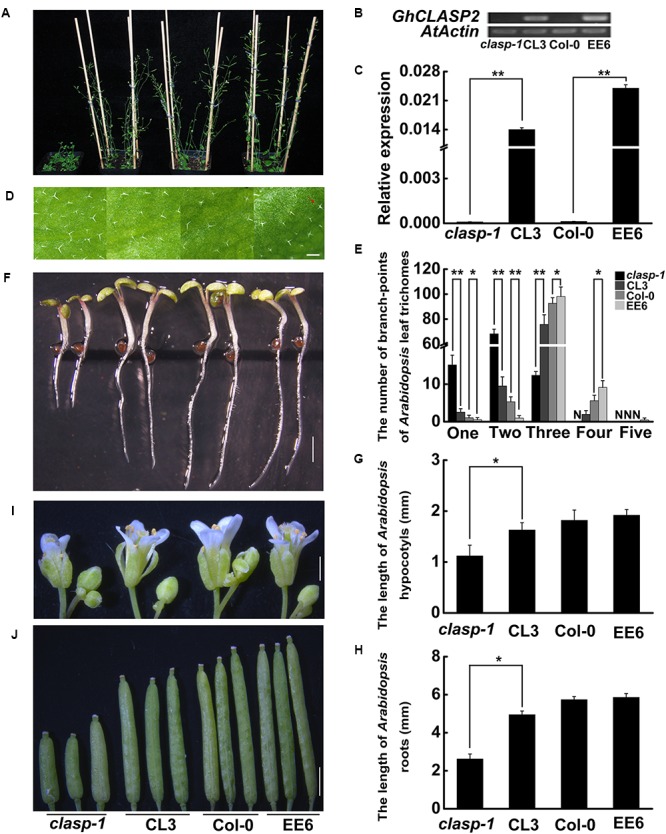
The phenotypes of *clasp-1*, complementing line (CL3), wild-type (Col-0), and the transgenic line with ectopic expression of *GhCLASP2* (EE6) in *Arabidopsis*. **(A)** Thirty-two-day-old plants. **(B)** sqPCR analysis of *GhCLASP2* expression in the plants shown in **(A)**. **(C)** qRT-PCR analysis of *GhCLASP2* expression in the plants shown in **(A)**. **(D)** Leaf trichomes of the four types of *Arabidopsis* plants. The red arrow indicates a leaf trichome with five branches (Bar = 200 μm). **(E)** The graph depicts the number of trichomes with different numbers of branch-points per leaf shown in **(D)** (*n* = 30). N, none; one, one branch; two, two branches; three, three branches; four, four branches; five, five branches in per leaf. **(F)** Seven-day-old *Arabidopsis* seedlings. Bar = 1 mm. **(G)** Hypocotyls length of the 7-day-old *Arabidopsis* seedlings shown in **(F)** (*n* = 20). **(H)** Root length of the 7-day-old *Arabidopsis* seedlings shown in **(F)** (*n* = 20). **(I)** Thirty-two-day-old *Arabidopsis* flowers (Bar = 1 mm). **(J)** Thirty-two-day-old *Arabidopsis* pods (Bar = 1 mm). For both sqPCR and qRT-PCR, *AtActin* was used as a reference gene. Data are means ± SD. *P*-values were determined based on Student’s *t*-tests. ^∗^ and ^∗∗^ indicate significant differences at *p* < 0.05 and *p* < 0.01, respectively.

To understand the function of *GhCLASP2*, we tried to complement the defective phenotypes of the *Arabidopsis* mutant *clasp-1* by transforming a copy of *GhCLASP2*, which was driven by the *Ubiquitin* promoter. For comparison, transgenic *Arabidopsis* plants with overexpressed *GhCLASP2* were also generated (**Figure 3**). *Arabidopsis* leaf trichomes have variable number of branches. In the wild-type, most trichomes (88.56%) had three branches. In contrast, most trichomes of *clasp-1* had two branches (71.26%). *clasp-1* also had more one-branch trichomes than the wild-type (**Figures 3D,E** and Supplementary Table 4). These observations were consistent with previous findings (Ambrose et al., 2007; Kirik et al., 2007). Compared to *clasp-1*, the complementary lines (CL3, CL6, and CL7) restored the phenotype of dominant three-branch trichomes (82.52–84.37%), although the percentage of three-branch trichomes was still not as high as that of the wild-type. These results suggested that *GhCLASP2* could rescue the defective leaf trichome phenotype of *clasp-1*. In the *GhCLASP2*-overexpressed lines, as in the wild-type, the majority leaf trichomes had three branches (89.80–91.41%), but the percentage was higher than that of the wild-type (88.56%). Furthermore, trichomes with four branches were significantly higher in the *GhCLASP2*-overexpressed lines than in the wild-type (**Figures 3D,E** and Supplementary Table 4). Trichomes with five branches were also observed in some *GhCLASP2*-overexpressed lines (Supplementary Table 4). These results suggest that the ectopic expression of *GhCLASP2* promoted trichome branching in *Arabidopsis* leaves and that trichome branching is positively correlated with the expression level of *GhCLASP2* (**Figures 3B,C**).

In addition, we investigated hypocotyl elongation and root growth in the same materials (**Figures 3F–H**). Compared to *clasp-1*, the three complementary lines had significantly elongated hypocotyls (1.50–1.63 mm versus 1.12 mm) and roots (4.95–5.01 mm versus 2.62 mm). However, transgenic lines with overexpressed-*GhCLASP2* and wild-type had a similar length of hypocotyls and roots (**Figures 3F–H** and Supplementary Table 5). Furthermore, *GhCLASP2* was able to rescue the defective phenotypes of flower and silique of *clasp-1*, although *Arabidopsis* lines overexpressing *GhCLASP2* and wild-type had similar flowers and siliques (**Figures 3I,J**). Together, these results suggest that *GhCLASP2* functions similarly as *AtCLASP* in plant growth and development.

### Subcellular Localization of GhCLASP2

**FIGURE 4:**
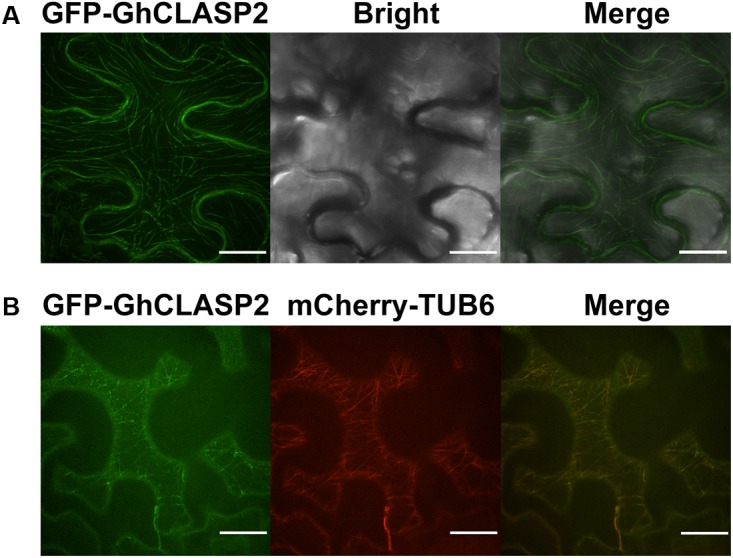
GFP-GhCLASP2 co-localized with MTs in the epidermal pavement cell of tobacco leaf. **(A)** Transiently expressed GFP-GhCLASP2 was localized in all MT arrays and induced stable MT bundles (Bar = 20 μm). **(B)** GFP-GhCLASP2 was co-expressed with a tubulin marker, mCherry-TUB6, in tobacco leaf epidermal cells (Bar = 25 μm).

To investigate the subcellular localization of GhCLASP2, we fused the full-length *GhCLASP2* coding sequence to the C terminus of the *GFP* gene driven by the 35S promoter (pGWB6-GFP-GhCLASP2) and infiltrated the construct into tobacco (*N. benthamiana*) leaf. Enriched GFP signal was observed along with the entire length of the filamentous structures in the leaf epidermal pavement cells (**Figure 4A**).

To investigate whether GhCLASP2 and MTs are co-localized, the pGWB6-GFP-GhCLASP2 construct and mCherry-TUB6, which labels β-tubulin, were transiently co-transformed into tobacco (*N. benthamiana*) leaf. GFP signal was found to be co-localized with MTs in the filamentous structures of pavement cells (**Figure 4B**). In the leaf epidermal cells, GFP-GhCLASP2 distributed in a nonuniform manner along the full length of MTs and seemed to be induced by extensive MT bundling (**Figure 4**). Just like AtCLASP, GhCLASP2 was prominently observed as small and immobile spots with variable intensity at MTs, indicating that GhCLASP2 interacts with MTs potentially through regulating MTs as a +TIP (Akhmanova et al., 2001; Wittmann and Waterman-Storer, 2005).

### *GhCLASP2* Is Required for the Growth and Development of Cotton

**FIGURE 5:**
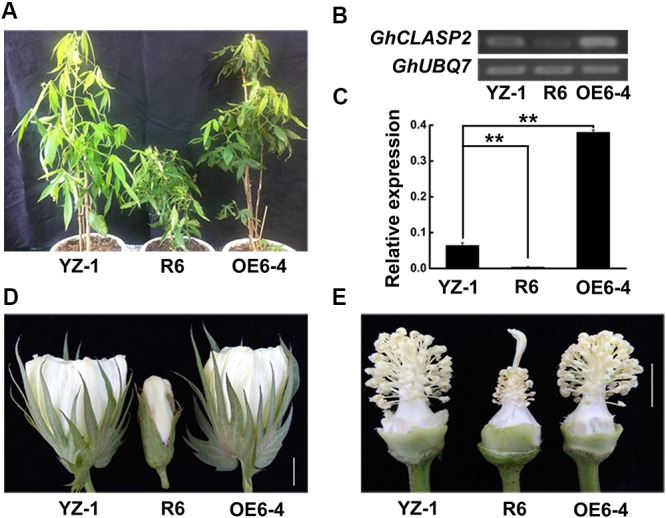
*GhCLASP2* affected cotton plant growth and development. **(A)** From left to right, the control plant YZ-1, the *GhCLASP2*-RNAi transgenic plant (R6), and the *GhCLASP2*-overexpressed transgenic plant (OE6-4). **(B)** sqPCR analysis of *GhCLASP2* expression in the plants shown in **(A)**. **(C)** qRT-PCR analysis of *GhCLASP2* expression in the plants shown in **(A)**. **(D)** Flower of YZ-1, R6, and OE6-4 on the anthesis day (0 DPA) (Bar = 1 cm). **(E)** Anther, pistil, and stamen from 0 DPA flower of YZ-1, R6, and OE6-4 (Bar = 1 cm). For both sqPCR and qRT-PCR, *GhUBQ7* was used as a reference gene. Data are means ± SD. *P*-values were determined based on Student’s *t*-tests. ^∗^ and ^∗∗^ indicate significant differences at *p* < 0.05 and *p* < 0.01, respectively.

**FIGURE 6:**
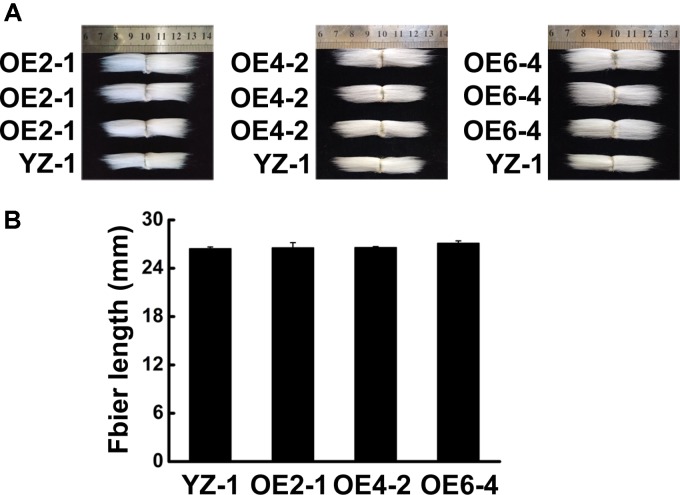
Phenotype of fiber in *GhCLASP2*-overexpressed transgenic cotton lines. **(A)** Phenotype of final mature fiber in *GhCLASP2*-overexpressed transgenic cotton lines (OE2-1, OE4-2, and OE6-4), comparing with YZ-1. **(B)** Average fiber length from YZ-1- and *GhCLASP2*-overexpressed transgenic cotton lines (OE2-1, OE4-2, and OE6-4). Data are means ± SD. Error bars indicate standard deviation of triplicate experiments.

To know the function of *GhCLASP2* in cotton development, we generated transgenic cotton plants with the *GhCLASP2*-overexpressed construct driven by the *Ubiquitin* promoter or the RNAi-*GhCLASP2* construct driven by the 35S promoter (Supplementary Figures 6A, 7A). Six *GhCLASP2*-overexpressed T_0_ transgenic lines were obtained. T_3_ homozygous transgenic lines were identified and used in further investigation. The up-regulated level of *GhCLASP2* in these plants was confirmed by sqPCR and qRT-PCR (**Figures 5B,C** and Supplementary Figure 6). Compared to the wild-type YZ-1, none of the *GhCLASP2*-overexpressed transgenic lines showed significant differences in both vegetative and reproductive growth (**Figure 5**). However, *GhCLASP2*-overexpressed lines had significantly increased fiber strength (3.85–4.67%) compared to the wild-type YZ-1 (**Table 1**), although they had a similar fiber length and micronaire value (**Figure 6**).

**Table 1:** Analysis of fiber quality between field-grown *GhCLASP2*-overexpressed transgenic lines and controls.

Lines	Fiber length (mm)	Fiber strength (cN ⋅ tex^-1^)	Micronaire value
YZ-1	26.43 ± 0.21	24.43 ± 0.06	5.80 ± 0.20
OE6-4	27.10 ± 0.30	25.37 ± 0.25^∗^	6.00 ± 0.01
OE4-2	26.57 ± 0.12	25.40 ± 0.50^∗^	5.80 ± 0.17
OE2-1	26.53 ± 0.65	25.57 ± 0.12^∗^	6.10 ± 0.20

*Fiber samples were harvested from field-grown T_*3*_ transgenic cotton. Values are mean ± standard deviation of assays for samples of three individual repeats from each line. YZ-1, the control line; OE6-4, OE4-2, and OE2-1, three different *GhCLASP2*-overexpressed transgenic lines. Asterisks indicate statistically significant differences between transgenic lines and the control plants YZ-1, as determined by the Student’s *t*-test. ^∗^ and ^∗∗^ indicate significant differences at *p* < 0.05 and *p* < 0.01, respectively*.

The *GhCLASP2*-specific down-regulation in the six *GhCLASP2*-RNAi T_0_ transgenic lines was confirmed by sqPCR and qRT-PCR (**Figures 5B,C** and Supplementary Figure 7). We did not detected down-regulation of other *GhCLASP2* homologs in the T_0_ transgenic lines (Supplementary Figure 8). The plant height of the T_0_ *GhCLASP2*-RNAi lines was shorter than that of the wild-type YZ-1 due to shorter internodes (**Figure 5**). In addition, the *GhCLASP2*-RNAi lines showed smaller flower, aborted anthers (without pollen grains), slightly elongated stigma, and sterility (**Figures 5A,D,E**). These phenotypes were similar to those observed in the *Arabidopsis* mutant *clasp-1* (Ambrose et al., 2007; Kirik et al., 2007). CLASP, acting as a MAP in cell, is involved in cell division and expansion (Ambrose et al., 2007). Due to the reduced expression level of *GhCLASP2* in the *GhCLASP2*-RNAi transgenic cotton plants, the stability of MTs was probably decreased, which affected the characteristics of cell wall and formation of cell cytoskeleton to cause the abnormal phenotypes observed in the *GhCLASP2*-RNAi lines.

### Transcriptional Changes of Genes Related to Fiber Development in *GhCLASP2*-Overexpressed Transgenic Cotton

**FIGURE 7:**
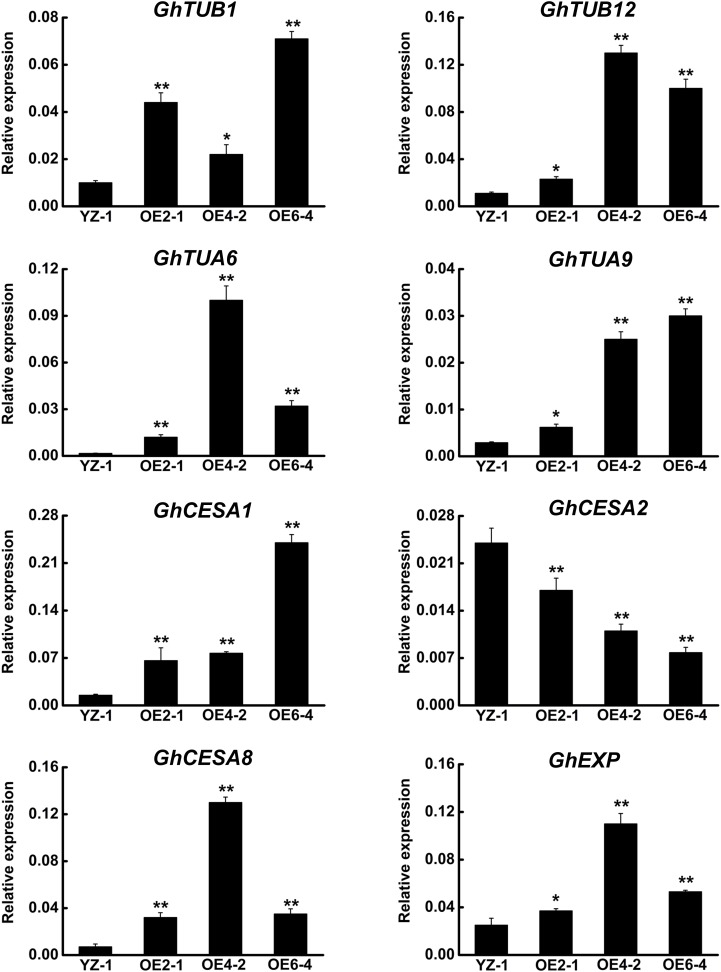
qRT-PCR analysis of fiber development-related genes in the *GhCLASP2*-overexpressed transgenic cotton lines. Fiber of 27DPA from the wild-type YZ-1 and three transgenic lines was used. Data are means ± SD. Error bars represent the standard deviation of triplicate experiments, and *GhUBQ7* was used as an internal control. *P*-values were determined based on Student’s *t*-tests. ^∗^ and ^∗∗^ indicate significant differences at *p* < 0.05 and *p* < 0.01, respectively.

Because *GhCLASP2*-overexpressed transgenic plants showed increased fiber strength, we analyzed the expression levels of eight selected genes related to fiber development in those plants by qRT-PCR (Supplementary Table 1) (Li et al., 2002, 2013; Xu et al., 2013). Compared to the wild-type, all three *GhCLASP2*-overexpressed lines showed an elevated level of MT-associated genes, i.e., *GhTUB1*, *GhTUB12*, *GhTUA6*, and *GhTUA9*, although the increased levels were gene and line dependent (**Figure 7**), suggesting that *GhCLASP2* might have the ability to induce transcription of MT-associated genes and to promote development of MTs, and finally development of cotton fibers.

Microtubules are intimately associated with cellulose synthesis activity, especially during secondary wall deposition (Wasteneys, 2004; Panteris and Galatis, 2005; Paredez et al., 2006). The secondary wall thickening of cotton fiber is mainly a process of cellulose synthesis and precipitation, which is crucial to the quality of cotton fibers, especially fiber strength. Because the expression level of *GhCLAP2* was rapidly increased in the developmental stage of secondary wall thickening (after 27 DPA), we examined the expression levels of three cellulose synthase genes (*CESAs*) in *GhCLASP2*-overexpressed transgenic lines. Compared to the wild-type, *GhCLASP2*-overexpressed transgenic lines had a higher expression level of *GhCESA1* and *GhCESA8*, but a lower expression level of *GhCESA2* (**Figure 7**). Since *AtCLASP* could be involved in cell expansion and division (Ambrose et al., 2007), we also analyzed the expression level of *GhEXP* in cotton fiber, which showed an increased level in all three transgenic lines, particularly in OE4-2 (**Figure 7**). These data suggest that overexpression of *GhCLASP2* may alter the expression level of genes related to fiber development and consequently promotes cotton fiber development.

## Discussion

Cytoplasmic linker-associated proteins are one of the MAP families that interact with and regulate MTs, an essential component of plant cytoskeleton. Genes encoding CLASPs have been bioinformatically identified in several species (Gardiner, 2013) but only the *Arabidopsis* CLASP has been functionally characterized so far (Ambrose et al., 2007; Kirik et al., 2007). In this work, we identified cotton *CLASP* genes and investigated the function of one of the eight *CLASPs* identified in *G*. *hirsutum*, *GhCLASP2*.

Upland cotton (*G*. *hirsutum*) is an allotetraploid and thought to have originated from a relatively recent interspecific hybridization event between an A genome-like ancestral species similar to *G*. *arboreum* or *G*. *herbaceum* and a D-genome-like species similar to *G*. *raimondii*. Each of the two diploid cotton species, *G*. *arboreum* and *G*. *raimondii*, contains four *CLASP* genes. All of the eight genes from the two diploids have been retained in *G*. *hirsutum*, suggesting conservation of the *CLASP* genes during cotton evolution. One reason for this conservation could be that these genes are essential for normal cotton development. Functional conservation of CLASPs is further supported by our observation that the *GhCLASP2*-RNAi cotton lines showed similar phenotypes as those of the *Arabidopsis clasp-1* mutant (**Figure 5**) and that the defective phenotypes of *clasp-1* could be rescued by *GhCLASP2* (**Figure 3**).

Microtubules play an important role in plant cell morphology and development (Hamada, 2014). It has been shown that MTs participated in the initiation and localization of epidermal protrusions (Mathur and Chua, 2000). Mutations in the factors regulating MTs caused misassembly of α/β tubulin dimers resulted in disruption of the formation of new MTs and the branching pattern of leaf trichomes (Kirik et al., 2002). Leaf trichomes of *Arabidopsis* plants overexpressing *GhCLASP2* had more branch numbers than the wild-type (**Figure 3**). GhCLASP2 was also co-localized with MTs (**Figure 4**). Therefore, like AtCLASP, GhCLASP2 might achieve its role through interacting with and regulating MTs.

Cotton fiber is an ideal model for studying cell development (Guan et al., 2014). In view of the similarity between cotton fiber development and *Arabidopsis* leaf trichome development (Machado et al., 2009). We reasoned that *CLASPs* would have a role in cotton fiber formation. Cellulose is the main component of cotton fiber, which accounts for more than 90% of dry weight of mature cotton fiber cells (Meinert and Delmer, 1977). Cellulose is synthesized by cellulose synthase complexes, mainly in the form of microfibrils (Kudlicka and Brown, 1997; Doblin et al., 2002). At the onset of secondary wall thickening, the rate of cellulose synthesis increases significantly. Studies have shown that *GhCESA1*, *GhCESA2*, and *GhCESA8* are mainly participating in secondary wall formation of cotton fiber. Cortical MTs are associated with cellulose synthase complexes and correlated with accumulation of cellulose microfibrils, especially during the period of secondary wall deposition (Gardiner et al., 2003; Wasteneys, 2004; Panteris and Galatis, 2005; Smith and Oppenheimer, 2005; Paredez et al., 2006). An involvement of cortical MTs in the movement of the cellulose synthase complex is evident in the plasma membrane (Mcfarlane et al., 2014). Secondary wall thickening of cotton fibers is crucial for the formation of cotton fiber quality, such as fiber fineness, maturity, and strength. We found that the expression level of *GhCLASP2* in cotton fibers increased dramatically from 24 DPA to 27 DPA and stayed high thereafter (**Figure 2**), and that the *GhCLASP2*-overexpressed lines had stronger fibers than the wild-type (**Figure 6** and **Table 1**). These results suggest a potential relationship between the expression level of *GhCLASP2* and fiber strength. We found that the expression levels of three cellulose synthase genes (*GhCESA1*, *GhCESA2*, and *GhCESA8*) have altered expression levels in the 27 DPA fibers of *GhCLASP2*-overexpressed transgenic cotton plants (**Figure 7**). Previous studies have shown that AtCLASP, as a MT binding protein, is most likely to be involved in cell expansion by affecting the mechanical properties of the cell wall, and thus affecting the organ morphology and axial extension in the plants (Ambrose et al., 2007). A gene encoding expansion (EXP) protein and four MT-associated genes (*GhTUA6*, *GhTUA9*, *GhTUB1*, and *GhTUB12*) showed up-regulated expression in the 27 DPA fibers of the *GhCLASP2*-overexpressed transgenic cotton plants (**Figure 7**). Based on these results, we speculate that increased fiber strength observed in the *GhCLASP2*-overexpressed plants is a result of coordinated up-regulation and down-regulation of a number of genes (such as *TUAs*, *TUBs*, *CESAs*, and expansin) involved in fiber development. It indicates that *GhCLASP2* has direct or indirect relationship with these genes. We thus propose that *GhCLASP2* might be involved in regulating fiber development, particularly fiber strength, by interacting with MTs and affecting cellulose biosynthesis and deposition. The network involving *GhCLAP2* that participates in fiber development is needed to be further investigated and analyzed in the future. *GhCLASP2* was relatively highly expressed in stem (**Figure 2**). Downregulation of *GhCLASP2* caused dwarfism due to shortened internode length, suggesting that cell division and/or elongation of stem were affected by the reduced expression level of *GhCLASP2*. This function of CLASP has been reported in *Arabidopsis*, where shorter and wider epidermal cells were observed in the hypocotyls of *clasp-1* (Ambrose et al., 2007). Cotton fiber is an extremely elongated single cell. We were unable to analyze the effect of knock-down *GhCLASP2* on fiber elongation, as all transgenic plants harboring the *GhCLASP2*-RNAi construct generated in this study were sterile and failed to produce any cotton boll; however, we did not observe fiber length difference between the plants overexpressing *GhCLASP2* and the wild-type (**Figure 6** and **Table 1**). Whether *CLASP* having negative effects on fiber cell elongation is yet to be investigated using other *CLASP* genes, such as *Gh_A08G1710/Gh_D08G2066*, that are preferentially expressed in cotton fibers and very weakly expressed in flowers.

There are eight *CLASPs* in *G. hirsutum*. Like *GhCLASP2*, some of them were also predominantly expressed in fibers, such as the At sub-genome homoeolog (*Gh_A07G1847*) of *GhCLASP2* and *Gh_A08G1710/Gh_D08G2066*. In this study we were unable to address their redundant roles in fiber development due to sterility of the *GhCLASP*-RNAi lines, but demonstrated the functional conservativeness between *GhCLASP2* and *AtCLASP*. In addition, our results suggest that it might be possible to improve cotton fiber strength by overexpressing *CLASPs*.

## Author Contributions

S-HZ, FX, Q-HZ, and JS conceived and designed the experiments. S-HZ and L-JZ performed the experiments. S-HZ analyzed the data. JS, Y-JL, FL, X-YZ, and Y-QS contributed reagents, materials, and analysis tools. S-HZ and Q-HZ wrote the paper.

## Conflict of Interest Statement

The authors declare that the research was conducted in the absence of any commercial or financial relationships that could be construed as a potential conflict of interest. The reviewer HM and the handling Editor declared their shared affiliation.

## Footnotes

1 https://www.cottongen.org/

2 http://www.ebi.ac.uk/Tools/msa/clustalw2/

3 http://www.expasy.org/tools

4 https://phytozome.jgi.doe.gov/pz/portal.html#!info?alias=Org_Ghirsutum_er
