# Supplementary material for: Identification and Functional Characterization of a Microtubule-Associated Protein, GhCLASP2, From Upland Cotton (Gossypium hirsutum L.)
Source: Front Plant Sci. 2018 Jun 27;9:882. doi: 10.3389/fpls.2018.00882 (PMC6030384; doi:10.3389/fpls.2018.00882)
Supplement: Supplementary file 1 [file Data_Sheet_1.pdf]

Supplementary Material

Identification and functional characterization of a microtubule associated protein, GhCLASP2, from upland cotton (*Gossypium hirsutum* L.)

Shou-Hong Zhu, Fei Xue, Yan-Jun Li, Feng Liu, Xin-Yu Zhang, Lan-Jie Zhao, Yu-Qiang Sun, Qian-Hao Zhu*, Jie Sun*

* **Correspondence:** Jie Sun: sunjie@shzu.edu.cn; Qian-Hao Zhu: qianhao.zhu@csiro.au.

1.1 Supplementary Tables

Supplementary Table 1. Oligonucleotides used for qRT-PCR in this study.

Primer name	Sequence(5'-3')
GhCLASP2-F	TGCCAGTAAGCACAATGCACTACA
GhCLASP2-R	AGGGCAAGCTCTCTGATTGAGA
Gh_A07G1847-F	TGCCAGTAAGCACAATGCACTTCT
Gh_A07G1847-R	AGGGCAAGCTCTCTGATTGAGGA
Gh_D08G2066-F	ACGTCCTTTTCTTCTGGGATGAGT
Gh_D08G2066-R	CACCAGAGGTACTTGATTCTAGTCCTAA
Gh_A08G1710-F	GAGGAGCTGCTGATTATCCATGTTTT
Gh_A08G1710-R	TGGAATGAAGATCTCAGCACATGCT
Gh_A07G1448-F	AGCCAAGAGTTCTTCAAGGGAGA
Gh_A07G1448-R	TGCTTGACCTTCTATCTGATAACTGTGC
Gh_D07G1541-F	AGCCAAGAGTTCTTCAAGGGAGT
Gh_D07G1541-R	TGCTTGACCTTCTATCTGATAACTGTGT
Gh_A09G0520-F	TGAAGCAACTTGTTGCACCTCTTT
Gh_A09G0520-R	AATCACAACCAGCTTGAAAAGGACC
GhCLASP1-F	AACCGAAGGTTTTCAGTTTCAATAGGCACA
GhCLASP1-R	GATGACAAAAGCTTCAACTCGCTGT
Gh_A07G1448/*Gh_D07G1541-F*	ATAGGCGGCATGCTTCTCCA
Gh_A07G1448/*Gh_D07G1541-R*	AGAGTACGCTCAGCACCTTTACCT
Gh_A09G0520/*GhCLASP1-F*	AGCGGCATTCTAAAGCTGTGGA
Gh_A09G0520/*GhCLASP1-R*	TTTGCTGCCTAAGAATTCGCCTT
Gh_A08G1710/*Gh_D08G2066-F*	TTCTGGAGAGATGAAGCCTGCAAT
Gh_A08G1710/*Gh_D08G2066-R*	TGGCAGCAATGCGTACAGAC
UBQ7-F	GAAGGCATTCCACCTGACCAAC

UBQ7-qRT-R	CTTGACCTTCTTCTTCTTGTGCTTG
Actin-qRT-F	GGTAACATTGTGCTCAGTGGTGG
Actin-qRT-R	AACGACCTTAATCTTCATGCTGC
GhTUB1F	CGGTACCATGGATAGCGTAA
GhTUB1R	TCCCTTAGCCCAATTGTTTC
GhTUB12F	AAACCTTATTCCATTCCCTCG
GhTUB12R	TCACCCTCCTGAACATCTCTTG
GhTUA6F	CATCAAGACCAAGCGCACAAT
GhTUA6R	CATTCAATCAATGGAGTTCCT
GhTUA9F	GTGGTGATGTTGTTCCCAAGGAC
GhTUA9R	AATACACCCAACAAAAATATCAAG
GhCESA1F	CCTCAACAAAGGGTACGAAGC
GhCESA1R	CAACACTGACCAAAGGACAACA
GhCESA2F	GTAGACACCAACTTCACCGTAACA
GhCESA2R	GCCACGACTCCGACCATGT
GhCESA8F	CCGGCATCTCAGATGCCATA
GhCESA8R	TTGAAGCCAATAGCACTGACC
GhEXPF	AGTCGAACCATAACCGTGACAGCC
GhEXPR	CCCAATTTCTGGACATAGGTAGCC

Supplementary Table 2. Oligonucleotides used for gene cloning and vector construction in this study.

Primer name	Sequence(5'-3')	Underlined	Destination
GhCLASP2-full-F	ATGGAGGAGGTACTGGAGT	-	*GhCLASP2* gene cloning
GhCLASP2-full-R	TTCATGACCAGCATCTAT	-	
GFP-*GhCLASP2*-F	caccATGGAGGAGGCACTGGAGT	adaptor	GFP vector
GFP-*GhCLASP2*- R	TTCATGACCAGCATCTAT	-	
GhCLASP2-OE-F	GGGGTACCATGGAGGAGGCACTGGAGT	*Kpn*I	Overexpression vector
GhCLASP2-OE-R	CGGGATCC TTCATGACCAGCATCTAT	*Bam*HI	
GhCLASP2-RNAi-F	caccAATCCCTTGGTAAAGGTGCTGG	adaptor	RNAi vector
GhCLASP2-RNAi-R	TGTCGCATTAGTCCTATATGATAACTCT	-	
Guslinker-F	CATGAAGATGCGGACTTACG	-	Identification of *GhCLASP2*-RNAi transgenic lines
GhCLASP2-RNAi-R	GGCTGCCTAACCAACTCCTT	-	
UBQTIN-F	TGGAACTGTATGTGTGTGTCA	-	Identification of *GhCLASP2*-overexpressed transgenic lines
GhCLASP2-R	TCGTTGGGGAAGAAACCTCCA	-	
NPT Ⅱ-F	GAGGCTATTCGGCTATGACTG	-	*NPT Ⅱ* detection
NPT Ⅱ-R	ATCGGGAGCGGCGATACCGTA	-	

Supplementary Table 3 Characteristics of CLASPs from diploid and allotetraploid cotton.

Sequence number	genomic sequence(bp)	protein length(aa)	CDS (bp)	Mw(kD)	PI
Cotton_A_35954	12203	1386	4161	153.81	6.55
Cotton_A_34471	11437	1409	4230	156.19	6.45
Cotton_A_33457	9982	1435	4308	158.89	6.69
Cotton_A_05890	9416	1436	4311	158.65	6.8
Gorai.001G183300.1	16540	1432	4299	158.59	6.79
Gorai.001G235400.1	10061	1439	4320	159.11	6.71
Gorai.006G054600.1	12641	1395	4188	154.93	6.52
Gorai.004G223500.1	10620	1435	4308	158.83	7.00
Gh_A07G1448	11469	1409	4230	156.08	6.38
Gh_D07G1541	11552	1432	4299	158.6	6.79
Gh_A09G0520	13086	1386	4161	153.84	6.62
Gh_D08G2066	9984	1435	4308	158.87	7.00
Gh_A08G1710	9994	1460	4383	161.81	6.76
Gh_A07G1847	9426	1439	4320	158.91	7.17
Gh_D07G2054	9335	1464	4395	162.32	7.16
GhCLASP1	NC	1395	4188	155.13	6.39
GhCLASP2	NC	1439	4320	158.95	6.79

NC, not clone.

Supplementary Table 4 The numbers of different branches of per leaf in *Arabidopsis* plant.

Lines		One branch	Two branches	Three branches	Four branches	Five branches
Mutant *clasp-1* (n=30)		15.14 ±2.60	68.24 ±3.72	12.38 ±0.98	0	0
Complementary of plant lines	CL3 (n=30)	2.55 ±0.91**	9.52 ±2.43**	75.8 ±7.67**	1.97 ±0.98	0
	CL6 (n=30)	3.07 ±0.75**	10.45 ±2.08**	73.72 ±5.97**	2.10 ±1.05	0
	CL7 (n=30)	2.17 ±1.00**	9.75 ±2.46**	74.41 ±6.37**	2.14 ±0.95	0
Wild type (Col-0) (n=30)		1.07 ±0.65	5.31 ±1.31	92.66 ±4.46	5.59 ±1.47	0
Ectopic expression of *GhCLASP2* lines	EE2(n=30)	0.66 ±0.55*	0.86 ±0.74**	97.21 ±8.08	7.10 ±1.29*	0.52 ±0.51
	EE5(n=30)	0.59 ±0.57*	0.90 ±0.67**	99.1 ±6.82*	8.10 ±1.88*	0.34 ±0.48
	EE6(n=30)	0.55 ±0.51*	0.97 ±0.63**	98.14 ±7.58*	9.14 ±1.81*	0.48 ±0.51

CL, complementary of plant lines; EE, ectopic expression of *GhCLASP2* lines. Data are means ±SD (n=30). P values are from Student's t tests. * and ** indicate significant differences at $p < 0.05$ and $p < 0.01$, respectively.

Supplementary Table 5. The length of the roots and hypocotyls in *Arabidopsis*.

Lines		Length of roots (mm)	Length of hypocotyls （mm）
Mutant *clasp-1* (n=20)		2.62 ±0.26	1.12 ±0.21
Complementary of plant lines	CL3 (n=20)	4.95 ±0.19*	1.63 ±0.14*
	CL6 (n=20)	5.01 ±0.17*	1.57 ±0.19*
	CL7 (n=20)	4.91 ±0.24*	1.50 ±0.15*
Wild type (Col-0) (n=20)		5. 74 ±0.16	1.82 ±0.20
Ectopic expression of *GhCLASP2* lines	EE2 (n=20)	5.87 ±0.21	1.81 ±0.15
	EE5 (n=20)	5.71 ±0.23	1.84 ±0.14
	EE6 (n=20)	5.86 ±0.20	1.92 ±0.11

CL, complementary of plant lines; EE, ectopic expression of *GhCLASP2* lines. Data are means ±SD (n=20). P values are from Student’s t tests. * and ** indicate significant differences at $p < 0.05$ and $p < 0.01$, respectively.

1.2 Supplementary Figures

```
Gohir.1Z037600.1 ATGGAGAAACTCCTTGAACTAGCACGTTCCAAGGACCCAAAGGACCGAGCAACAGGGGCT      60
GhCLASP1         ATGGAGAAACTCCTTGAACTAGCACGTTCCAAGGACCCAAAGGACCGAGCAACAGGGGCT      60
CotAD_68468      ATGGAGAAACTCCTTGAACTAGCACGTTCCAAGGACCCAAAGGACCGAGCAACAGGGGCT      60
                 ************************************************************

Gohir.1Z037600.1 GAGCTTCTTCATTCTCTTCTCCAGTCTTCCACTGGACCCTTGTCTTCTTCTGACGTTGAA      120
GhCLASP1         GAGCTTCTTCATTCTCTTCTCCAGTCTTCCACTGGACCCTTGTCTTCTTCTGACGTTGAA      120
CotAD_68468      GAGCTTCTTCATTCTCTTCTCCAGTCTTCCACTGGACCCTTGTCTTCTTCCGACGTTGAA      120
                 ************************************************** *********

Gohir.1Z037600.1 TCTCTTGTTTCAGCTTGTCTTCACCTTCTCAATGACCCATCTAACTTAAACGCCTCTTTG      180
GhCLASP1         TCTCTTGTTTCAGCTTGTCTTCACCTTCTCAATGACCCATCTAACTTAAACGCCTCTTTG      180
CotAD_68468      TCTCTTGTTTCAACTTGTATTCACCTTCTCAATGACCCATCTAACTTAAACGCCTCTTTG      180
                 ************ ***** *****************************************

Gohir.1Z037600.1 GGAGCTCTCCAGTGTCTAGCATCTGCTGCCGTGCTCTCAGCTGACAACTTGAAGCTGCAT      240
GhCLASP1         GGAGCTCTCCAGTGTCTAGCATCTGCTGCCGTGCTCTCAGCTGACAACTTGAAGCTGCAT      240
CotAD_68468      GGAGCTCTCCAGTGTCTAGCATCTGCTGCCGTGCTCTCAGCTGACAACTTGAAGCTGCAT      240
                 ************************************************************

Gohir.1Z037600.1 TTTGATGGGGTTTTACCTGCAATTGTGGAGTGCTTAGGGGATGATGAGCAGCCTTTACGT      300
GhCLASP1         TTTGATGGGGTTTTACCTGCAATTGTGGAGTGCTTAGGGGATGATGAGCAGCCTTTACGT      300
CotAD_68468      TTTGATGGGGTTTTACCTGCAATTGTGGAGTGTTTAGGGGATAATAAGCAGCCTTTACGT      300
                 ******************************** ********* ** **************

Gohir.1Z037600.1 GATGCAGCTAGAGGGCTGTTGTTGACTTTCATGGAGGTTTCTTCTCCAACTATCATTGTA      360
GhCLASP1         GATGCAGCTAGAGGGCTGTTGTTGACTTTCATGGAGGTTTCTTCTCCAACTATCATTGTA      360
CotAD_68468      GATGCAGCTAGAGGGCTGTTGTTGACTTTCATGGAGGTTTCTTCTCCAGCTATTATTGTA      360
                 ************************************************ **** ******

Gohir.1Z037600.1 GATAAAGTAGGGCCGATAACCTGGGCGCATAACAGCCCGAGAGTTCGTGAAGAGTTTATG      420
GhCLASP1         GATAAAGTAGGGCCGATAACCTGGGCGCATAACAGCCCGAGAGTTCGTGAAGAGTTTATG      420
CotAD_68468      GATAAAGTTGGGCCGATAACCTGGGCGCATAACAGCCTGAGAGTTCGTGAAGAGTTTATG      420
                 ******** **************************** **********************

Gohir.1Z037600.1 AGGACCGTCACTTCTGCTATCACTGTTTTTACGTCGATGGAGTTCATGAAGGCTATTCTT      480
GhCLASP1         AGGACCGTCACTTCTGCTATCACTGTTTTTACGTCGATGGAGTTCATGAAGGCTATTCTT      480
CotAD_68468      AGGACTGTCACTTCTGCTATCACTGTTTTTACGTCGATGGAGTTCATGAAGGCTATTCTT      480
                 ***** ******************************************************
```

```
Gohir.1Z037600.1 CCTCCGATTTTACAGATGTTGAATGACTCAATTCAGAGCATTAGAGAACGTGCTTCATTG    540
GhCLASP1         CCTCCGATTTTACAGATGTTGAATGACTCAATTCAGAGCATTAGAGAACGTGCTTCATTG    540
CotAD_68468      CCTCCGATTTTACAGATGTTGAATGACTCAATTCAGAGCATTAGAGAAGCTGCTACATTG    540
                 ************************************************  **** *****

Gohir.1Z037600.1 TGCATTGAGG--------------------------------------------------    550
GhCLASP1         TGCATTGAGG--------------------------------------------------    550
CotAD_68468      TGCATTGAGGTGTGGTTTGGTGTCTCCAATCACCTCCGCGGCTCTGTTGTTTCTGGCCTA    600
                 **********

Gohir.1Z037600.1 -------AAATGTACATGCAGTTTGGGCCTGAATTTCTTGCTGAACTCCAGCGAAACAAT    603
GhCLASP1         -------AAATGTACATGCAGTTTGGGCCTGAATTTCTTGCTGAACTCCAGCGAAACAAT    603
CotAD_68468      GATCAAGAAATGTACATGCAGTTTGGGCCTGAATTTCTTGCTGAACTCCAGCGAAACAAT    660
                        *****************************************************

Gohir.1Z037600.1 CTTCCTTCATCAGTGTTAGGAGATATTAATATCAGATTGCAGAAGATAGAACCGAAGGTT    663
GhCLASP1         CTTCCTTCATCAGTGTTAGGAGATATTAATATCAGATTGCAGAAGATAGAACCGAAGGTT    663
CotAD_68468      CTTCCTTCATCAGTGTTAGGAGATATTAATATCAGATTGCAGAAGATAGAACCGAAGGTT    720
                 ************************************************************

Gohir.1Z037600.1 TTCAGTTTCAATAGACACATGTCATCTGCTTCTTCTGATCAAGTTGACTCAGCCAAGGGG    723
GhCLASP1         TTCAGTTTCAATAGGCACATGTCATCTGCTTCTTCTGATCAAGTTGACTCAGCCAAGGGG    723
CotAD_68468      TTCAGTTTCAATAGACACGTGTCATCTGCTTCTTCTGATCAAGTTGACTCAGCCAAGGGG    780
                 ************** *** *****************************************

Gohir.1Z037600.1 AACCAAGCAGAGAGCAATCCTAAGACAAAGGATTCTTTTAAAGATATATCTGTTTCTGGA    783
GhCLASP1         AACCAAGCAGAGAGCAATCCTAAGACAAAGGATTCTTTTAAAGATATATCTGTTTCTGGA    783
CotAD_68468      AACCAAGCAGAGAGCAATCCTAAGACAAAGGATTCTTTTAAAGATATATCTGTTTCTGGA    840
                 ************************************************************

Gohir.1Z037600.1 GGAGAGATTGACTTTACAGACAAACCCATAGTTCCAGTTACTTTAAACTCTGAAAAGGAG    843
GhCLASP1         GGAGAGATTGACTTTACAGACAAACCCATAGTTCCAGTTACTTTAAACTCTGAAAAGGAG    843
CotAD_68468      GGAGAGATTGACTTTACAGACAAACCCATAGTTCCAGTTACTTTAAACTCTGAAAAGGAG    900
                 ************************************************************

Gohir.1Z037600.1 CTAATAAGAGAAATGGAGAAGATTGCGTGTATGCTTGTAGCGGAAAATGATTGGTCTGTT    903
GhCLASP1         CTAATAAGAGAAATGGAGAAGATTGCGTGTATGCTTGTAGCGGAAAATGATTGGTCTGTT    903
CotAD_68468      CTAATAAGAGAAATGGAGAAGATTGCGTGTATGCTTGTAGCGGAAAATGATTGGTCTGTT    960
                 ************************************************************

Gohir.1Z037600.1 CGAATAGCTGCTATGCAGCGAGTTGAAGCTTTTGTCATCGGAGGTGCCACCAGTTATCCA    963
GhCLASP1         CGAATAGCTGCTATACAGCGAGTTGAAGCTTTTGTCATCGGAGGTGCCACCAGTTATCCA    963
CotAD_68468      CGAATAGCTGCTATGCAGCGAGTTGAAGCTCTTGTCATTGGAGGTGCCACCAGTTATCCA    1020
                 ************** *************** ******* *********************
```

```
Gohir.1Z037600.1 TGCTTTCATGCTCTCTTGAAGCAACTTGTTGCACCTCTTCGCACACAGTTGTCCGACCGA    1023
GhCLASP1         TGCTTTCATGCTCTCTTGAAGCAACTTGTTGCACCTCTTCGCACACAGTTGTCCGACCGA    1023
CotAD_68468      TGTTTTCATGCTCTCTTGAAGCAACTTGTTGCACCTCTTTGCACACAGTTGTCCGACCGA    1080
                 ** ************************************ ********************

Gohir.1Z037600.1 AGGTCTAGCATTGTAAAGCAGGCTTGTCATCTCTTATGTTTCTTGTCAAAAGAGCTGTTG    1083
GhCLASP1         AGGTCTAGCATTGTAAAGCAGGCTTGTCATCTCTTATGTTTCTTGTCAAAAGAGCTGTTG    1083
CotAD_68468      AGGTCTAGCATCGTAAAGCAGGCCTGTCATCTCTTATGTTTCTTGTCAAAAGAGCTGTTG    1140
                 *********** *********** ************************************

Gohir.1Z037600.1 GGGGATTTTGAAGCTTGTTCGGAAATGTTCATCCCAGTCCTTTTCAAGCTGGTTGTGATT    1143
GhCLASP1         GGGGATTTTGAAGCTTGTTCGGAAATGTTCATCCCAGTCCTTTTCAAGCTGGTTGTGATT    1143
CotAD_68468      GGGGATTTTGAAGCTTGTTCGGAAATGTTCATCCCGGTCCTTTTCAAGCTGGTTGTGATT    1200
                 *********************************** ************************

Gohir.1Z037600.1 ACGGTTCTTATAATAGCTGAATCCGCAGACACCTGCATCAAAACAATGCTGCGTAACTGC    1203
GhCLASP1         ACGGTTCTTATAATAGCTGAATCCGCAGACACCTGCATCAAAACAATGCTGCGTAACTGC    1203
CotAD_68468      ACGGTTCTTATAATAGCTGAATCCGCAGACACCTGCATCAAAACAATGCTGCGTAACTGC    1260
                 ************************************************************

Gohir.1Z037600.1 AAAGTTTCCCGTGTACTTCCTCAAATAGTTAATCATGCAAAACATGACCGTAATGCAGTA    1263
GhCLASP1         AAAGTTTCCCGTGTACTTCCTCAAATAGTTAATCATGCAAAACATGACCGTAATGCAGTA    1263
CotAD_68468      AAAGTTTCCCGTGTACTTCCTCAAATAGTTAACCATGCAAAACATGACCGTAATGCAGTA    1320
                 ******************************** ***************************

Gohir.1Z037600.1 CTCCGTGCTAGATGCTGTGAGTACTCTCTTCTGATGCTGGAGTACTGGGCCGATGCGCCA    1323
GhCLASP1         CTCCGTGCTAGATGCCGTGAGTACTCTCTTCTGATGCTGGAGTACTGGGCCGATGCGCCA    1323
CotAD_68468      CTCCGTGCTAGATGCTGTGAGTACTCTCTTCTGATGCTGGAGTACTGGGCCTATGCACCA    1380
                 *************** *********************************** **** ***

Gohir.1Z037600.1 GAAATACAGAAATCTGCTGATCTTTACGAGGATCTTATAAAATGCTGTATAGCTGATGCA    1383
GhCLASP1         GAAATACAGAAATCTGCTGATCTTTACGAGGATCTTATAAAATGCTGCATAGCTGATGCA    1383
CotAD_68468      GAAATACAGAAATCTGCAGATCTTTACGAGGATCTTATAAAATGCTGTGTAGCTGATGCA    1440
                 ***************** *****************************  ***********

Gohir.1Z037600.1 ATGAGTGAGGTACAGTCAAATGCTCGTCGTTGCTACAGACTGTTCAAGAAAACTTGGCCA    1443
GhCLASP1         ATGAGTGAGGTACAGTCAAATGCTCGTCGTTGCTACAGACTGTTCAAGAAAACTTGGCCA    1443
CotAD_68468      ATGAGTGAGGTACGGTCAAATGCTCGTCGTTGCTACAGACTGTTCAAGAAAACTTGGCCA    1500
                 ************* **********************************************

Gohir.1Z037600.1 GAGCGTTCTCAGCAACTATTTCTGTCCTTTGATTCAGTCGTCCAAAGGATGATAAACGAT    1503
GhCLASP1         GAGCGTTCTCAGCAACTATTTCTGTCCTTTGATTCAGTCGTCCAAAGGATGATAAACGAT    1503
CotAD_68468      GAGCGTTCTCAGCAACTATTTCTGTCCTTTGATTCAGTCGTCCAAAGGATGATAAACGAT    1560
                 ************************************************************
```

```
Gohir.1Z037600.1 GAAGATGGGACCGCGCATAGACGCCATCCTTCTCCTTCAGTTCGAGTAAGGGATGTCCGT    1563
GhCLASP1         GAAGATGGGACCGCGCATAGACGCCATCCTTCTCCTTCAGTTCGAGTAAGGGATGTCCGT    1563
CotAD_68468      GAAGATGGGACCACACATAGACGCCATCCTTCTCCTTCAGTTCGAGTAAAGGATGTCCGT    1620
                 ************ * ********************************** **********

Gohir.1Z037600.1 AAATCCCATAAGGCTTTTCAAGCACCTACTTCTACAACTTTACCTGGACCTGCAACTTCA    1623
GhCLASP1         AAATCCCATAAGGCTTTTCAAGCACCTACTTCTACAACTTTACCTGGACCTGCAACTTCA    1623
CotAD_68468      AAATCCCATAAGGCTTTTCAAGCACCTACTTCTACAACTTTACCTGGACCTGCAACTTCA    1680
                 ************************************************************

Gohir.1Z037600.1 GCAGTTACCACAGTGGATCGGAATAGGGCTGTACCAGGAGGCATTCCTCTATCTGCTGGT    1683
GhCLASP1         GCAGTTACCACAGTGGATCGGAATAGGGCTGTACCAGGAGGCATTCCTCTATCTGCTGGT    1683
CotAD_68468      GCAGTTACCACAGTGGATCGGAATAGGGCTGTACCAGGAGGCATTCCTCTATCTGCTGGT    1740
                 ************************************************************

Gohir.1Z037600.1 CCATTTCTCTCTGAAGTAAAGTCACATCGAAATGGTACTGAAAGAAGTTTTGAAAACATG    1743
GhCLASP1         CCATTTCTCTCTGAAGTAAAGTCACATCGAAATGGTACTGAAAGAAGTTTTGAAAACATG    1743
CotAD_68468      CCATTTCTCTCTGAAGTAAAGTCACATCGAAATGGTACTGAAAGAAGTTTTGAAAACATG    1800
                 ************************************************************

Gohir.1Z037600.1 CTGCATGCAAACAAACAGCGGGCCCCGGCTATCGAAAATGCGCTTGCTGGTTTAAATATA    1803
GhCLASP1         CTGCATGCAAACAAACAGCGGGCCCCGGCTATCGAAAATGCGCTTGCTGGTTTAAATATA    1803
CotAD_68468      CCAAATACAAACAAACAGCGGGCTCCGGCTATCGAAAATGCGCTTGCTGGTTTAATTATA    1860
                 *   ** **************** ******************************* ****

Gohir.1Z037600.1 TCTGAGAAACTCAGTTATCAAAAGAAGCGGTCAGCTAGCTTTGATTTAGGAGTTGACCCT    1863
GhCLASP1         TCTGAGAAACTCAGTTATCAAAAGAAGCGGTCAGCTAGCTTTGATTTAGGAGTTGACCCT    1863
CotAD_68468      TCTGAAAAACTCAGTTATCAAAAGAAGAGGTCAGCTAGCTTTGATTTAGGAGTTGACCCT    1920
                 ***** ********************* ********************************

Gohir.1Z037600.1 CCTCCAGCATGTGGCCTCTCATTTCCCCCTATTGTCGCTGCTTCAGCTGTTACTGCAAAT    1923
GhCLASP1         CCTCCAGCATGTGGCCTCTCATTTCCCCCTATTGTCGCTGCTTCAGCTGTTACTGCAAAT    1923
CotAD_68468      CCTCCAGCTTGTGGCCTCTCATTTCCCCCTATTGTCGCTGCTTCAGCTGTTACTGCAAAT    1980
                 ******** ***************************************************

Gohir.1Z037600.1 GCTATTTTTACTGACTCAACTGCGTCAACTGTTAAAGCTTGTTTGTCATCGAAATCAGGG    1983
GhCLASP1         GCTATTTTTACTGACTCAACTGCGTCAACTGTTAAAGCTTGTTTGTCATCGAAATCAGGG    1983
CotAD_68468      GCTATTTTTACTGACTCAACTGCATCGACCGTTAAAGCTTGTTTGTCATCGAAATCAGGG    2040
                 *********************** ** ** ******************************

Gohir.1Z037600.1 AAGCAAGCTTGTGGTTTTATGGAAGAGTATTCTGATGATAGGTTTGCCCGGAAATCGGTA    2043
GhCLASP1         AAGCAAGCTTGTGGTTTTATGGAAGAGTATTCTGATGATAGGTTTGCCCGGAAATCGGTA    2043
CotAD_68468      AAGCAAGCTTGTGGTTTTATGGAAGAGCATTCTGATGATAGGTTTGCCCGGAAATCCGTA    2100
                 *************************** **************************** ***
```

```
Gohir.1Z037600.1 GATAAGCATATTGACAAACAGTATGTAGAGACTTCTTCAAAAGATGCCAATTTTAGGGAC     2103
GhCLASP1         GATAAGCATATTGACAAACAGTATGTAGAGACTTCTTCAAAAGATGCCAATTTTAGGGAC     2103
CotAD_68468      GATAAGCATATTGACAAACAGCATGTAGATACTTCTTCAAAAGATGCCAATTTTAGGGAC     2160
                 ********************* ******* ******************************

Gohir.1Z037600.1 TTCCAAGGTAATATTATTCCAAACTTCCAGAGGCCGCTTTTAAGAAAGAACATTTCAACT     2163
GhCLASP1         TTCCAAGGTAATATTATTCCAAACTTCCAGAGGCCGCTTTTAAGAAAGAACATTTCAACT     2163
CotAD_68468      TTCCAAGGTAATATTATTCCAAACTTCCAGAGGCCGCTTTTAAGAAAGAACATTTCAACT     2220
                 ************************************************************

Gohir.1Z037600.1 CGAGTTTCTGGAAGCAGTAGAAGCTTTCTAGATGACAATCAGATACTACTTAGTGAAATG     2223
GhCLASP1         CGAGTTTCTGGAAGCAGTAGAAGCTTTCTAGATGACAATCAGATACTACTTAGTGAAATG     2223
CotAD_68468      CGAGTTTCTGGAAACAGTAGAAGTTTTCTAGATGACAATCAGATACTACTTAGTGAAATG     2280
                 ************* ********* ************************************

Gohir.1Z037600.1 TCAAACTATGTGGACGGCCCAGCATCACTCCAGGAGGCTCTGACAGAGGGTCTCAGTCCA     2283
GhCLASP1         TCAAACTATGTGGACGGCCCAGCATCACTCCAGGAGGCTCTGACAGAGGGTCTCAGTCCA     2283
CotAD_68468      TCGAACTATGTGGATGGCCCAGCATCACTCCAGGAGGCTCTGACAGAGGGTCTCAGTCCA     2340
                 ** *********** *********************************************

Gohir.1Z037600.1 AATTCCAATTGGTCCGCTAGAGTTGCGGCTTTTAATTATGTCCGAAGTCTGCTGCAGCAG     2343
GhCLASP1         AATTCCAATTGGTCCGCTAGAGTTGCGGCTTTTAATTATGTCCGAAGTCTGCTGCAGCAG     2343
CotAD_68468      AATTCTAATTGGTCTGCTAGAGTTGCGGCTTTTAATTATGTCCGAAGTCTGCTGCAGCAG     2400
                 ***** ******** *********************************************

Gohir.1Z037600.1 GACCAAAAAGGCGTGCAAGAAGTTGCACAGAATTTTGAGAAAGTCATGAAGTTGTTTTTC     2403
GhCLASP1         GACCAAAGAGGCGTGCAAGAAGTTGCACAGAATTTTGAGAAAGTCATGAAGTTGTTTTTC     2403
CotAD_68468      GACCAAAAAGGAGTCCAAGAAGTTGCACAGAATTTTGAGAAAGTCATGAAGTTGTTTTTC     2460
                 ******* *** ** *********************************************

Gohir.1Z037600.1 CAACACTTGGATGATCCACACCATAAAGTTGCACACGCCGCTCTCTCGGCCCTTGCAGAT     2463
GhCLASP1         CAACACTTGGGTGATCCACACCATAAAGTTGCACACGCCGCTCTCTCGGCCCTTGCAGAT     2463
CotAD_68468      CAACACTTGGATGATCCGCACCATAAAGTTGCACACGCCGCTCTCTCGGCCCTTGCAGAT     2520
                 ********** ****** ******************************************

Gohir.1Z037600.1 ATAATTTCAGCATGTAGAAAACCTTTTGAAAGTTACTTGGATCGAATCTTACACCATGTG     2523
GhCLASP1         ATAATTTCAGCATGTAGAAAACCTTTTGAAAGTTACTTGGATCGAATCTTACACCATGTG     2523
CotAD_68468      ATAATTTCAGCATGTAGAAAACCTTTTGAAAGTTACTTGGATCGAATCTTACACCATGTG     2580
                 ************************************************************

Gohir.1Z037600.1 TTTTCAAGGTTAATTGATCCGAAGGAATCAGTTCGGCAGCTTTCTGTAATGAATCTGGAA     2583
GhCLASP1         TTTTCAAGGTTAATTGATCCGAAGGAATCAGTTCGGCAGCTTTCTGTAATGAATCTGGAA     2583
CotAD_68468      TTTTCAAGGTTAATTGATCCGAAGGAATCAGTTCGGCAGCTTTCTGTAATGAATCTGGAA     2640
                 ************************************************************
```

```
Gohir.1Z037600.1 GTTGTAAGCAAAGCCTATGGCATAGATTCTCTTTTACCAGCTTTGCTCCGTGCATTAGAT    2643
GhCLASP1         GTTGTAAGCAAAGCCTATGGCATAGATTCTCTTTTACCAGCTTTGCTCCGTGCATTAGAT    2643
CotAD_68468      GTTGTAAGCAAAGCCTATGGCATAGATTCTCTTTTACCAGCTTTGCTCCGTGCCTTAGAT    2700
                 ***************************************************** ******

Gohir.1Z037600.1 GAACAGAGATCACCGAAGGCAAAACTGGCTGTTATAGAATATGCTATCAGTTCCTTCAAG    2703
GhCLASP1         GAACAGAGATCACCGAAGGCAAAACTGGCTGTTATAGAATATGCTATCAGTTCCTTCAAG    2703
CotAD_68468      GAACAGAGATCACCGAAGGCAAAACTGGCTGTTATAGAATACGCTATCAGTTCTTTCAAG    2760
                 ***************************************** *********** ******

Gohir.1Z037600.1 AAGAATGCTATGAATTCAGACGGTGCTGCTAATAGCGGCATTCTAAAGCTGTGGATTGCT    2763
GhCLASP1         AAGAATGCTATGAATTCAGACGGTGCTGCTAATAGCGGCATTCTAAAGCTGTGGATTGCT    2763
CotAD_68468      AAGAATGCTATGAATTCAGACGGTGCTGCTAATAGCGGCATTCTAAAGCTGTGGATTGCT    2820
                 ************************************************************

Gohir.1Z037600.1 AAATTAATACCTTTGATCTATGATAAAAACACAAAACTAAAAGAAGCAGCCATCTCCTGC    2823
GhCLASP1         AAATTAATACCTTTGATCTATGATAAAAACACAAAACTAAAAGAAGCAGCCATCTCCTGC    2823
CotAD_68468      AAATTAATACCTTTGATCTATGATAAAAACACAAAACTAAAAGAAGCAGCCATCTCCTGC    2880
                 ************************************************************

Gohir.1Z037600.1 ATTGTATCCGTACATACTCACTATGACTCCACTGGTGTTATAAACTACATCATGTGTATG    2883
GhCLASP1         ATTGTATCCGTACATACTCACTATGACTCCACTGGTGTTATAAACTACATCATGTGTATG    2883
CotAD_68468      ATTGTATCTATACATACTCACTATGACTCCGTTGGTGTTATAAACTACATCATGTGTATG    2940
                 ********  ********************  ****************************

Gohir.1Z037600.1 TCAGCTGAAGAGCAAAATTCTCTAAGGCGAATTCTTAGGCAGCAAACTCCTCGAATTGAA    2943
GhCLASP1         TCAGCTGAAGAGCAAAATTCTCTAAGGCGAATTCTTAGGCAGCAAACTCCTCGAATTGAA    2943
CotAD_68468      TCAGCTGAAGAGCAAAATTCTCTAAGGCGAATTCTTAGGCAGCAAACTCCTCGAATTGAA    3000
                 ************************************************************

Gohir.1Z037600.1 ATGGATCTTATGAACTTCTTGCAGAACAAGAGAGAAAGACCCCGTCCTAGAAACAGTCAC    3003
GhCLASP1         ATGGATCTTATGAACTTCTTGCAGAACAAGAGAGAAAGACCCCGTCCTAGAAACAGTCAC    3003
CotAD_68468      ATGGATCTTATGAACTTCTTGCAGAACAAGAGAGAAAGACCCCGTCCTAGAAACAGTCAC    3060
                 ************************************************************

Gohir.1Z037600.1 GAGCCATCTGATGTCGGACTTTCTCCTGATGGTGAATATACTGGTGCATTAAAGAAGGCT    3063
GhCLASP1         GAGCCATCTGATGTCGGACTTTCTCCTGATGGTGAATATACTGGTGCATTAAAGAAGGCC    3063
CotAD_68468      GAGCCATCTGATGTCGGACTTTCTCCTGATGGTGAATATACTGGTGCATTGAAGAAGGCT    3120
                 ************************************************** ******** 

Gohir.1Z037600.1 TACTATTTTGGAAGATATTCTTCGGGTTCGAATGATATCGATAACAGCAAGAAGTTGTTC    3123
GhCLASP1         TACTATTTTGGAAGATATTCTTCGGGTTCGAATGATATCGATAACAGCAAGAAGTTGTTC    3123
CotAD_68468      TACTATTTTGGAAGATATTCTTCAGGTTCGAATGATATCGATAACAGCAAGAAGTTGTTC    3180
                 *********************** ************************************
```

```
Gohir.1Z037600.1 TCCATGCAAGAACCGATGCAAGTTTTAGAACCTATTGCTCAAAAAGTATCAAGTGAAGCT    3183
GhCLASP1         TCCATGCAAGAACCGATGCAAGTTTTAGAACCTATTGCTCAAAAAGTATCAAGTGAAGCT    3183
CotAD_68468      TCCATGCAAGAACCGATGCAAGTTTTAGAACCTATTGCTCAAAAAGTATCAAGTGAAGCT    3240
                 ************************************************************

Gohir.1Z037600.1 CAGAAAAATTTACCTCAGAATCTCGAAGGTGGCTCTAATAAAGCTTTTACTTGCACTACT    3243
GhCLASP1         CAGAAAAATTTACCTCAGAATCTCGAAGGTGGCTCTAATAAAGCTTTTACTTGCACTACT    3243
CotAD_68468      CAGAAAAATTTACCTCAGAATCTCGAAGGCGGCTGTAATAATGCTTTTACTTGCACTACT    3300
                 ***************************** **** ****** ******************

Gohir.1Z037600.1 ACTGGTGCTGATGAAAATGTGGAATCCCCAATGAGCCATCCTGATGGTGTTGATTGTGAG    3303
GhCLASP1         ACTGGTGCTGATGAAAATGTGGAATCCCCAATGAGCCATCCTGATGGTGTTGATTGTGAG    3303
CotAD_68468      ACTGGTGCTGA------------------------CCATCCTGATGGTGTTGATTGTGAG    3336
                 ***********                        *************************

Gohir.1Z037600.1 AATTCAATGAACAACGACTGGCCTTCGTCATGTTCTGACATGAATACAATGACAATCTCT    3363
GhCLASP1         AATTCAATGAACAACGACTGGCCTTCGTCATGTTCTGACATGGATACAATGACAATCTCT    3363
CotAD_68468      AATTCAATGG---ACGACTGGCCTTCGACATGTTTTGACATGAATACGATGACAATCTCT    3393
                 *********    ************** ****** ******* **** ************

Gohir.1Z037600.1 AACCATTTTGGAGAAAGCAATTCCAATGTTGGTATTGACACTTTTGTTGATGTCAATAAT    3423
GhCLASP1         AACCATTTTGGAGAAAGCAATTCCAATGTTGGTATTGACACTTTTGTTGATGTCAATAAT    3423
CotAD_68468      AATCATTTTGGAATAAGCAATTCCACTATTGGTATTGACACTTTTGTTGATGTCAATAAT    3453
                 ** *********  *********** * ********************************

Gohir.1Z037600.1 AGTCATGAGAATTCCAGCAATACCAAGACTTGCTGTTCTCTAAAATCGCGTATCATCATT    3483
GhCLASP1         AGTCATGAGAATTCCAGCAATACCAAGACTTGCTGTTCTCTAAAATCGCGTATCATCATT    3483
CotAD_68468      AGTAATGAGAATTCCAGCAATACCAAGACTTGCTCTTCTCTAAAATCGCGTATCATCATT    3513
                 *** ****************************** *************************

Gohir.1Z037600.1 CCTCAAATTCTTCACCAGATTTCTAACAATAAGGAGAGTGCAACCATTAGACAGCAAGCA    3543
GhCLASP1         CCTCAAATTCTTCACCAGATTTCTAACAATAAGGAGAGTGCAACCATTAGACAGCAAGCA    3543
CotAD_68468      CCTCAAATTCTTCACCAGATTTCTAACAATAAGGAGAGTGCAACCGTTAGACAGCTAGCA    3573
                 ********************************************* ********* ****

Gohir.1Z037600.1 CTTCAGCAGCTCATAGAAGCTTCCATGGATACAAACCTTTCGATATGGACAAAGCATTTC    3603
GhCLASP1         CTTCAGCAGCTCATAGAAGCTTCCATGGATACAAACCTTTCGATATGGACAAAGCATTTC    3603
CotAD_68468      CTTCAGCAGCTCATAGAAGCTTCCATGGATACAAACCTTTCGTTATGGACAAAGCATTTC    3633
                 ****************************************** *****************

Gohir.1Z037600.1 AATCAGATTCTGAAAGTTGTCATTGAGGTGTTGGACGATCCTGATTCTTCAACCCGGGAA    3663
GhCLASP1         AATCAGATTCTGAAAGTTGTCATTGAGGTGTTGGACGATCCTGATTCTTCAACCCGGGAA    3663
CotAD_68468      AACCAGATTCTGAAAGTTATCATTGAGGTGTTGGATGCTCCTGATTCTTCAACCCAGGAA    3693
                 ** *************** **************** * ***************** ****
```

```
Gohir.1Z037600.1 TTGACCTTGCAGTTAGTAGCTGACATGGTGAACAAACAGAAAGATGCGATGGAAGATTCT     3723
GhCLASP1         TTGACCTTGCAGTTAGTAGCTGACATGGTGGACAAACAGAAAGATGCGATGGAAGATTCT     3723
CotAD_68468      TTGACCCTGCAGTTAGTAGCTGACATGGTGAACAAACAGAAAGATGCGATGGAAGATTCT     3753
                 ****** *********************** *****************************

Gohir.1Z037600.1 ATTGAAATTGTAATTGAAAAGTTACTTCATATTGCTAAGGATGCAGTTTCCAAGGTTTCA     3783
GhCLASP1         ATTGAAATTGTAATTGAAAAGTTACTTCATATTGCTAAGGATGCAGTTTCCAAGGTTTCA     3783
CotAD_68468      ATTGAAATTGTAATTGCAAAGTTACCTCATATCGCTAAGGATGCAGTTTCCAAGGTTTCA     3813
                 **************** ******** ****** ***************************

Gohir.1Z037600.1 ATTGAAGCAGAGAAATGCTTATCAATGATTTTATCAGAATATGACCGTTTCAAATGTCTA     3843
GhCLASP1         ATTGAAGCAGAGAAATGCTTATCAATGATTTTATCAGAATATGACCGTTTCAAATGTCTA     3843
CotAD_68468      ATTGAAGCAGAGAACTGCTTATCAATGATTTTATCAGAATATGACCCTTTCAAATGTCTA     3873
                 ************** ******************************* *************

Gohir.1Z037600.1 AGTGTTATTGTAGCCCTTTTAATCCATGAAGATGAGAAAACTCTTATTTTTTGCATCAAG     3903
GhCLASP1         AGTGATATTGTAGCCCTTTTAATCCATGAAGATGAGAAAACTCTTATTTTTTGCATCAAG     3903
CotAD_68468      AGTGTTATTGTAGCCCTTTTAATCCATGAAGATGAGAAAACTCTTATTTTTTGCATCAAG     3933
                 **** *******************************************************

Gohir.1Z037600.1 ACATTAACAAAGCTTGTCCATCGGCTTTCTCAAGAGGAACTTCTGGCTCAGCTACCTTCA     3963
GhCLASP1         ACATTAACAAAGCCTGTCCATCGGCTTTCTCAAGAGGAACTTCTGGCTCAGCTACCTTCA     3963
CotAD_68468      ACATTAACAAAGCTTGTCCGTCGGCTTTCTCAAGAGGAACTTCTGGCTCAGCTACCTTCA     3993
                 ************* ***** ****************************************

Gohir.1Z037600.1 TTTTTACCGGTTCTTTTTTGCGCATTCAGCAATCAAAGCGCTGAAGTTCGCAAGA-----     4018
GhCLASP1         TTTTTACCGGTTCTTTTTTGCGCATTCAGCAATCAAAGCGCTGAAGTTCGCAAGA-----     4018
CotAD_68468      TTTTCACCAGTTCTTTTTTGCGCATTCAGCAATCAAAGCGCTGAAGTTCGCAAGATACTG     4053
                 **** *** **********************************************

Gohir.1Z037600.1 ------------------------------------------------------------     4018
GhCLASP1         ------------------------------------------------------------     4018
CotAD_68468      GTTTATGTCCCCATGTTTGAAAATGTGAGGGACATGGGTAATTCGAGAAAAATGAGAGTG     4113

Gohir.1Z037600.1 ----------------------------CTGTTGTTTTCTGCTTGGTCGACATTTACGTC     4050
GhCLASP1         ----------------------------CTGTTGTTTTCTGCTTGGTCGACATTTACGTC     4050
CotAD_68468      GCCTTTGAACTTAGATACGTAGGGCATACTGTTGTTTTCTGCTTGGTCGATATTTACGTC     4173
                                             ********************** *********

Gohir.1Z037600.1 ATGCTCGGGAAGGCGTTCCGGCCATACCTGCAGGATCTCAATGGGACCCAGTTGCGTCTG     4110
GhCLASP1         ATGCTCGGGAAGGCGTTCCGGCCATACCTGCAGGATCTCAATGGGACCCAGTTGCGTCTG     4110
CotAD_68468      ATGCTCGGGAAGGCGTTCCGGCCATACTTGCAGGATCTCAATGGGACCCAGTTGCGTCTG     4233
                 ************************** *********************************
```

```
Gohir.1Z037600.1 GTGACTCTTTATGCCAACAGAATTTCTCAAGCCAGAACTGGAACTTGTGTTGAAGCTATT     4170
GhCLASP1         GTGACTCTTTATGCCAACAGAATTTCTCAAGCCAGAACTGGAACTTGTGTTGAAGCTATT     4170
CotAD_68468      GTGACTCTTTATGCCAACAGAATTTCTCAAGCCAGAACTGGAACTTGTGTTGAAGCTATT     4293
                 ************************************************************

Gohir.1Z037600.1 AATTTGCCAGCAGTCTGA                                               4188
GhCLASP1         AATTTGCCAGCAGTCTGA                                               4188
CotAD_68468      AATTTGCCGGCAGTCTGA                                               4311
                 ******** *********
```

Figure 1 Multiple sequence alignment of *GhCLASP1*, *CotAD_68468* and *Gohir.1Z037600.*

```
CotAD_04861        ATGGAGGAGGTACTGGAGTTAGCACGTGCCAAAGACACCAAGGAGCGGATGGCGGCGGTG    60
Gh_D07G2054        ATGGAGGAGGTACTGGAGTTAGCACGTGCCAAAGACACCAAGGAGCGGATGGCGGCGGTG    60
GhCLASP2           ATGGAGGAGGTACTGGAGTTAGCACGTGCCAAAGACACCAAGGAGCGGATGGCGGCGGTG    60
Gohir.D07G208500   ATGGAGGAGGTACTGGAGTTAGCACGTGCCAAAGACACCAAGGAGCGGATGGCGGCGGTG    60
                   ************************************************************

CotAD_04861        GAGCGGCTCTACCAACTCCTTGAAGGCTCTAGAAAGAGTCTCACTTCTTCGGAAGTCACT    120
Gh_D07G2054        GAGCGGCTCTACCAACTCCTTGAAGGCTCTAGAAAGAGTCTCACTTCTTCGGAAGTCACT    120
GhCLASP2           GAGCGGCTCTACCAACTCCTTGAAGGCTCTAAAAAGAGTCTCACTTCTTCGGAAGTCACT    120
Gohir.D07G208500   GAGCGGCTCTACCAACTCCTTGAAGGCTCTAGAAAGAGTCTCACTTCTTCGGAAGTCACT    120
                   ******************************* ****************************

CotAD_04861        TCGCTTGTTGACTGCTGCTTGGATCTCCTGAAGGACAACAATTTTAGAGTCTCTCAGGGA    180
Gh_D07G2054        TCGCTTGTTGACTGCTGCTTGGATCTCCTGAAGGACAACAATTTTAGAGTCTCTCAGGGA    180
GhCLASP2           TCGCTCGTTGACTGCTGCTTGGATCTCCTCAAGGACAACAATTTTAGAGTCTCTCAGGGA    180
Gohir.D07G208500   TCGCTCGTTGACTGCTGCTTGGATCTCCTCAAGGACAACAATTTTAGAGTCTCTCAGGGA    180
                   ***** *********************** ******************************

CotAD_04861        GCTCTCCAGGCTCTTGCTTCCGCCGCCGTTCTTTCCGGTGATCACTTGAAGTTGCACTTC    240
Gh_D07G2054        GCTCTCCAGGCTCTTGCTTCCGCCGCCGTTCTTTCCGGTGATCACTTGAAGTTGCACTTC    240
GhCLASP2           GCTCTCCAGGCTCTTGCTTCCGCCGCCGTTCTTTCCGGTGATCACTTGAAGTTGCACTTC    240
Gohir.D07G208500   GCTCTCCAGGCTCTTGCTTCCGCCGCCGTTCTTTCCGGTGATCACTTGAAGTTGCACTTC    240
                   ************************************************************

CotAD_04861        AACGCTCTCGTTCCTGCGGTTGTTGAACGGTTAGGTGACGCCAAACAACCTGTTAGAGAC    300
Gh_D07G2054        AACGCTCTCGTTCCTGCGGTTGTTGAACGGTTAGGTGACGCCAAACAACCTGTTAGAGAC    300
GhCLASP2           AACGCTCTCGTTCCTGCGGTTGTTGAACGGTTAGGTGACGCCAAACAACCTGTTAGAGAC    300
Gohir.D07G208500   AACGCTCTCGTTCCTGCGGTTGTTGAACGGTTAGGTGACGCCAAACAACCTGTTAGAGAC    300
                   ************************************************************

CotAD_04861        GCTGCCAGGCGCCTACTGCTCACTCTCATGGAGGTTTCTTCCCCAACGATTATTGTTGAA    360
Gh_D07G2054        GCTGCCAGGCGCCTACTGCTCACTCTCATGGAGGTTTCTTCCCCAACGATTATTGTTGAA    360
GhCLASP2           GCTGCCAGGCGCCTACTGCTCACTCTCATGGAGGTTTCTTCCCCAACGATTATTGTTGAA    360
Gohir.D07G208500   GCTGCCAGGCGCCTACTGCTCACTCTCATGGAGGTTTCTTCCCCAACGATTATTGTTGAA    360
                   ************************************************************

CotAD_04861        AGAGCTGGCGCTTATGGCTGGACGCGTAGAAGTTGGAGAGTTCGGGAGGAATTTGCCCGG    420
Gh_D07G2054        AGAGCTGGCTCTTATGCCTGGACGCATAGAAGTTGGAGAGTTCGGGAGGAATTTGCCCGG    420
GhCLASP2           AGAGCTGGCTCTTATGCCTGGACGCATAGAAGTTGGAGAGTTCGGGAGGAATTTGCCCGG    420
Gohir.D07G208500   AGAGCTGGCTCTTATGCCTGGACGCATAGAAGTTGGAGAGTTCGGGAGGAATTTGCCCGG    420
                   ********* ****** ******** **********************************

CotAD_04861        ACAGTCACATCATCAATCAGTCTTTTTTCATCTACTGAACTTCCACTTCAACGGGCGATT    480
Gh_D07G2054        ACAGTCACATCATCAATCAGTCTTTTTTCATCTACTGAACTTCCACTTCAACGGGCGATT    480
```

```
GhCLASP2          ACAGTCACATCATCAATCAGTCTTTTTTCATCTACTGAACTTCCACTTCAACGGGCGATT      480
Gohir.D07G208500  ACAGTCACATCATCAATCAGTCTTTTTTCATCTACTGAACTTCCACTTCAACGGGCGATT      480
                  ************************************************************

CotAD_04861       CTTCCTCCTATTTTGCAGATGTTGAATGACACAAATCCTGGTGTTCGTGAAGCTGCTATA      540
Gh_D07G2054       CTTCCTCCTATTTTGCAGATGTTGAATGACACAAATCCTGGTGTTCGTGAAGCTGCTATA      540
GhCLASP2          CTTCCTCCTATTTTGCAGATGTTGAATGACACAAATCCTGGTGTTCGTGAAGCTGCTATA      540
Gohir.D07G208500  CTTCCTCCTATTTTGCAGATGTTGAATGACACAAATCCTGGTGTTCGTGAAGCTGCTATA      540
                  ************************************************************

CotAD_04861       TTATGCATCGAGGAAATGTATACGCAAGCCGGAACTCAATTTCGAGATGAACTTCATCGT      600
Gh_D07G2054       TTATGCATCGAGGAAATGTATACGCAAGCCGGAACTCAATTTCGAGATGAACTTCATCGT      600
GhCLASP2          TTATGCATCGAGGAAATGTATACGCAAGCCGGAACTCAATTTCGAGATGAACTTCATCGT      600
Gohir.D07G208500  TTATGCATCGAGGAAATGTATACGCAAGCCGGAACTCAATTTCGAGATGAACTTCATCGT      600
                  ************************************************************

CotAD_04861       CATCAGCTTCCTGGATCTATGATGAGAGATATTAATGCCAGACTAGAGAAAATTGAGCCA      660
Gh_D07G2054       CATCAGCTTCCTGGATCTATGATGAGAGATATTAATGCCAGACTAGAGAAAATTGAGCCA      660
GhCLASP2          CATCAGCTTCCTGGATCTATGATGAGAGATATTAATGCCAGACTAGAGAAAATTGAGCCA      660
Gohir.D07G208500  CATCAGCTTCCTGGATCTATGATGAGAGATATTAATGCCAGACTAGAGAAAATTGAGCCA      660
                  ************************************************************

CotAD_04861       CAAGTTCGACATTCAGATGGAACTTTGGGTGGTTTTGCTACCGGAGAGATAAAGCCTGCA      720
Gh_D07G2054       CAAGTTCGACATTCAGATGGAACTTTGGGTGGTTTTGCTACTGGAGAGATAAAGCCTGCA      720
GhCLASP2          CAAGTTCGACATTCAGATGGAACTTTGGGTGGTTTTGCTACTGGAGAGATAAAGCCTGCA      720
Gohir.D07G208500  CAAGTTCGACATTCAGATGGAACTTTGGGTGGTTTTGCTACTGGAGAGATAAAGCCTGCA      720
                  ***************************************** ******************

CotAD_04861       GTACGTAATCCCAAGAAAAGCAGTCCAAGAGCCAAGAGTTCTTCGAGGGAGACATCACTT      780
Gh_D07G2054       GTACGTAATCCCAAGAAAAGCAGTCCAAGAGCCAAGAGTTCTTCGAGGGAGACATCACTT      780
GhCLASP2          GTACGTAATCCCAAGAAAAGCAGTCCAAGAGCCAAGAGTTCTTCGAGGGAGACATCACTT      780
Gohir.D07G208500  GTACGTAATCCCAAGAAAAGCAGTCCAAGAGCCAAGAGTTCTTCGAGGGAGACATCACTT      780
                  ************************************************************

CotAD_04861       TTTGGAGGTGAAAGTGATATCACTGAAAAGCCAATAGATCCAATTCAAGTTTATTCAGAT      840
Gh_D07G2054       TTTGGAGGTGAAAGTGATATCACTGAAAAGCCAATAGATCCAATTCAAGTTTATTCAGAT      840
GhCLASP2          TTTGGAGGTGAAAGTGATATCACTGAAAAGCCAATAGATCCAATTCAAGTTTATTCAGAT      840
Gohir.D07G208500  TTTGGAGGTGAAAGTGATATCACTGAAAAGCCAATAGATCCAATTCAAGTTTATTCAGAT      840
                  ************************************************************

CotAD_04861       AAGGAGCTGGTAAGAGAATTTGAGAAAATTGCCTCTACTCTTGTGCCAGAAAAAGATTGG      900
Gh_D07G2054       AAGGAGCTGGTAAGGGAATTTGAGAAAATTGCCTCTACTCTTGTTCCAGAAAAAGATTGG      900
GhCLASP2          AAGGAGCTGGTAAGAGAATTTGAGAAAATTGCCTCTACTCTTGTGCCAGAAAAAGATTGG      900
Gohir.D07G208500  AAGGAGCTGGTAAGAGAATTTGAGAAAATTGCCTCTACTCTTGTGCCAGAAAAAGATTGG      900
```

```
                   ************** ***************************** ***************

CotAD_04861        TCCATACGCATTGCTGCCATGCAGAGACTTGAAGGGCTTGTTTCTGGAGGTGCTGCTGAT     960
Gh_D07G2054        TCCATACGCATTGCTGCCATGCAGAGACTTGAAGGGCTTGTTTCTGGAGGTGCTGCTGAT     960
GhCLASP2           TCCATACGCATTGCTGCCATGCAGAGACTTGAAGGGCTTGTTTCTGGAGGTGCTGCTGAT     960
Gohir.D07G208500   TCCATACGCATTGCTGCCATGCAGAGACTTGAAGGGCTTGTTTCTGGAGGTGCTGCTGAT     960
                   ************************************************************

CotAD_04861        TATCCGTCTTTTCGGGGACTCTTGAAGCAGCTTGTTGGCCCACTAAGTACACAGTTATCA    1020
Gh_D07G2054        TATCCGTGTTTTCGGGGACTCTTGAAGCAGCTTGTTGGCCCACTAAGCACACAGTTATCA    1020
GhCLASP2           TATCCGTGTTTTCGGGGACTCTTGAAGCAGCTTGTTGGCCCACTAAGCACACAGTTATCA    1020
Gohir.D07G208500   TATCCGTGTTTTCGGGGACTCTTGAAGCAGCTTGTTGGCCCACTAAGCACACAGTTATCA    1020
                   ******* *************************************** ************

CotAD_04861        GATAGGAGGTCAAGCATTGTGAAGCAGGCTTGCCATTTGTTATCCTTCTTATCAAAGGAG    1080
Gh_D07G2054        GATAGGAGGTCAAGCATTGTGAAGCAGGCTTGCCATTTGTTATCCTTCTTATCAAAGGAG    1080
GhCLASP2           GATAGGAGGTCAAGCATTGTGAAGCAGGCTTGCCATTTGTTATCCTTCTTATCAAAGGAG    1080
Gohir.D07G208500   GATAGGAGGTCAAGCATTGTGAAGCAGGCTTGCCATTTGTTATCCTTCTTATCAAAGGAG    1080
                   ************************************************************

CotAD_04861        CTCTTGGGAGATTTTGAGGCATGTGCTGAGATGTTCATCCCGGTACTTTTCAAGTTGGTT    1140
Gh_D07G2054        CTCTTGGGAGATTTTGAGGCATGTGCTGAGATGTTCATCCCAGTACTTTTCAAGTTGGTT    1140
GhCLASP2           CTCTTGGGAGATTTTGAGGCATGTGCTGAGATGTTCATCCCGGTACTTTTCAAGTTGGTT    1140
Gohir.D07G208500   CTCTTGGGAGATTTTGAGGCATGTGCTGAGATGTTCATCCCGGTACTTTTCAAGTTGGTT    1140
                   ***************************************** ******************

CotAD_04861        GTGATTACTGTGCTTGTAATTGCAGAGTCCGCAGATAACTGCATAAAAACAATGTTGCGT    1200
Gh_D07G2054        GTGATTACTGTGCTTGTAATTGCAGAGTCCGCAGATAACTGCATAAAAACAATGTTGCGT    1200
GhCLASP2           GTGATTACTGTGCTTGTAATTGCAGAGTCCGCAGATAACTGCATAAAAACAATGTTGCGT    1200
Gohir.D07G208500   GTGATTACTGTGCTTGTAATTGCAGAGTCCGCAGATAACTGCATAAAAACAATGTTGCGT    1200
                   ************************************************************

CotAD_04861        AACTGCAAAGTTGCCCGTGTGCTTCCCCGCATAGCTGATTGTGCAAAGAATGACCGTAGT    1260
Gh_D07G2054        AACTGCAAAGTTGCCCGTGTGCTTCCCCGCATAGCTGATTGTGCAAAGAATGACCGTAGT    1260
GhCLASP2           AACTGCAAAGTTGCCCGTGTGCTTCCCCGCATAGCTGATTGTGCAAAGAATGACCGTAGT    1260
Gohir.D07G208500   AACTGCAAAGTTGCCCGTGTGCTTCCCCGCATAGCTGATTGTGCAAAGAATGACCGTAGT    1260
                   ************************************************************

CotAD_04861        GCTGTACTCCGTGCCAGGT-----------------------------------------    1279
Gh_D07G2054        GCTGTACTCCGTGCCAGGTCTTCCTGGTTTTGCTATTTTATGAAGTCTTTTATTTGTTAT    1320
GhCLASP2           GCTGTACTCCGTGCCAGGT-----------------------------------------    1279
Gohir.D07G208500   GCTGTACTCCGTGCCAGGT-----------------------------------------    1279
                   *******************
```

```
CotAD_04861       ----------------------------------GTTGTGAATATGCATTATTGATACTT     1305
Gh_D07G2054       TTCTATATTCAACTTTTATATTCTTATTGCAGGTGTTGTGAATATGCATTATTGATACTT     1380
GhCLASP2          ----------------------------------GTTGTGAATATGCATTATTGATACTT     1305
Gohir.D07G208500  ----------------------------------GTTGTGAATATGCATTATTGATACTT     1305
                                                    **************************

CotAD_04861       GAACATTGGCCTGATGCACCAGAAATACAGAGATCGGCTGATTTATACGAGGATCTGATT     1365
Gh_D07G2054       GAACATTGGCCTGATGCACCAGAAATACAGAGATCGGCTGATTTATACGAGGATCTGATT     1440
GhCLASP2          GAACATTGGCCTGATGCACCAGAAATACAGAGATCGGCTGATTTATACGAGGATCTGATT     1365
Gohir.D07G208500  GAACATTGGCCTGATGCACCAGAAATACAGAGATCGGCTGATTTATACGAGGATCTGATT     1365
                  ************************************************************

CotAD_04861       AGGTGCTGTGTTGCTGATGCAATGAGTGAGGTACGATCAACTGCTAGAATGTGCTACAGA     1425
Gh_D07G2054       AGGTGCTGTGTTGCTGATGCAATGAGTGAGGTACGATCAACTGCTAGAATGTGCTACAGA     1500
GhCLASP2          AGGTGCTGTGTTGCTGATGCAATGAGTGAGGTACGATCAACTGCTAGAATGTGCTACAGA     1425
Gohir.D07G208500  AGGTGCTGTGTTGCTGATGCAATGAGTGAGGTACGATCAACTGCTAGAATGTGCTACAGA     1425
                  ************************************************************

CotAD_04861       ATGTTCTCCAAAACTTGGCCAGATCGTTCTCGTCGCTTGTTCACCTCCTTTGATCCTGCT     1485
Gh_D07G2054       ATGTTCTCCAAAACTTGGCCAGATCGTTCTCGTCGCTTGTTCACCTCCTTTGATCCTGCT     1560
GhCLASP2          ATGTTCTCCAAAACTTGGCCAGATCGTTCTCGTCGCTTGTTCACCTCCTTTGATCCTGCT     1485
Gohir.D07G208500  ATGTTCTCCAAAACTTGGCCAGATCGTTCTCGTCGCTTGTTCACCTCCTTTGATCCTGCT     1485
                  ************************************************************

CotAD_04861       ATTCAAAGGATAATTAACGAAGAAGATGGAGGGATGCATAGGCGGCATGCTTCTCCTTCT     1545
Gh_D07G2054       ATTCAAAGGATAATTAACGAAGAAGATGGAGGGATGCATAGGCGGCATGCTTCTCCTTCT     1620
GhCLASP2          ATTCAAAGGATAATTAACGAAGAAGATGGAGGGATGCATAGGCGGCATGCTTCTCCTTCT     1545
Gohir.D07G208500  ATTCAAAGGATAATTAACGAAGAAGATGGAGGGATGCATAGGCGGCATGCTTCTCCTTCT     1545
                  ************************************************************

CotAD_04861       GTCCGTGACAGAAATGTTAAAATGCCAATTAGCTCCCAATCTTCTAACAGTGCACATCTA     1605
Gh_D07G2054       GTCCGTGACAGAAATGTTAAAATGCCAATTAGCTCCCAATCTTCTGCCAGTGCACATCTA     1680
GhCLASP2          GTCCGTGACAGAAATGTTAAAATGCCAATTAGCTCCCAATCTTCTGCCAGTGCACATCTA     1605
Gohir.D07G208500  GTCCGTGACAGAAATGTTAAAATGCCAATTAGCTCCCAATCTTCTGCCAGTGCACATCTA     1605
                  *********************************************  *************

CotAD_04861       CCTGGATATCAAACATCTGCTATAGTTGCCATGGATAGAACTTCAACTTTATCCTCGGGC     1665
Gh_D07G2054       CCTGGATATCAAACATCTGCTATAGTTGCCATGGATAGAACTTCAACTTTATCCTCGGGC     1740
GhCLASP2          CCTGGATATCAAACATCTGCTATAGTTGCCATGGATAGAACTTCAACTTTATCCTCGGGC     1665
Gohir.D07G208500  CCTGGATATCAAACATCTGCTATAGTTGCCATGGATAGAACTTCAACTTTATCCTCGGGC     1665
                  ************************************************************

CotAD_04861       ACACCTCTCACATCTGGGTTGAATCTATCCCAATCAAAATCCCTTGGTAAAGGTGCTGGC     1725
Gh_D07G2054       ACACCTCTCACATCTGGGTTGAATCTATCCCAATCAAAATCCCTTGGTAAAGGTGCTGGC     1800
```

```
GhCLASP2          ACACCTCTCACATCTGGGTTGAATCTATCCCAATCAAAATCCCTTGGTAAAGGTGCTGGC     1725
Gohir.D07G208500  ACACCTCTCACATCTGGGTTGAATCTATCCCAATCAAAATCCCTTGGTAAAGGTGCTGGC     1725
                  ************************************************************

CotAD_04861       CGTACTTTGGAGAGTGTGTTGCATGCAAGCAAACAGAAAGTCAGTGCCATTGAAAGTATG     1785
Gh_D07G2054       CGTACTTTGGAGAGTGTGTTGCATGCAAGCAAACAGAAAGTCAGTGCCATTGAAAGTATG     1860
GhCLASP2          CGTACTTTGGAGAGTGTGTTGCATGCAAGCAAACAGAAAGTCAGTGCCATTGAAAGTATG     1785
Gohir.D07G208500  CGTACTTTGGAGAGTGTGTTGCATGCAAGCAAACAGAAAGTCAGTGCCATTGAAAGTATG     1785
                  ************************************************************

CotAD_04861       CTTAGAGGTCTGGACATATCCCAAAAACAGAGGTCAACAAGTTTGGATCTAGGAGTTGAC     1845
Gh_D07G2054       CTTAGAGGTCTGGACATATCCCAAAAACAGAGGTCAACAAGTTTGGATCTAGGAGTTGAC     1920
GhCLASP2          CTTAGAGGTCTGGACATATCCCAAAAACAGAGGTCAACAAGTTTGGATCTAGGAGTTGAC     1845
Gohir.D07G208500  CTTAGAGGTCTGGACATATCCCAAAAACAGAGGTCAACAAGTTTGGATCTAGGAGTTGAC     1845
                  ************************************************************

CotAD_04861       ------------------------------------------------------------     1845
Gh_D07G2054       CCTCCATCATCTCGTGATCCACCATTCCCTGCCGTTGTTCCAGCTTCTAATAGCCTCACA     1980
GhCLASP2          CCTCCATCATCTCGTGATCCACCATTCCCTGCCGTTGTTCCAGCTTCTAATAGCCTCACA     1905
Gohir.D07G208500  CCTCCATCATCTCGTGATCCACCATTCCCTGCCGTTGTTCCAGCTTCTAATAGCCTCACA     1905


CotAD_04861       ------------------------------------------------------------     1845
Gh_D07G2054       AGCTCTTTAGGACTAGAATCGACTACCTCTACTGTTGGTAAGGGTAGCAACCGCAATGGT     2040
GhCLASP2          AGCTCTTTAGGACTAGAATCGACTACCTCTACTGTTGGTAAGGGTAGCAACCGCAATGGT     1965
Gohir.D07G208500  AGCTCTTTAGGACTAGAATCGACTACCTCTACTGTTGGTAAGGGTAGCAACCGCAATGGT     1965


CotAD_04861       ------------------------------------------------------------     1845
Gh_D07G2054       GGTCTGATAATGTCTGACATAATTTCTCAAATTCAAGCTTCCAAAGATTCAGGAGAGTTA     2100
GhCLASP2          GGTCTGATAATGTCTGACATAATTTCTCAAATTCAAGCTTCCAAAGATTCAGGAGAGTTA     2025
Gohir.D07G208500  GGTCTGATAATGTCTGACATAATTTCTCAAATTCAAGCTTCCAAAGATTCAGGAGAGTTA     2025


CotAD_04861       ------------------------------------------------------------     1845
Gh_D07G2054       TCATATAGGACTAATGCGACAACCGAGTCTTTGCCAGCTTTCATATCATACTCTGCCAAG     2160
GhCLASP2          TCATATAGGACTAATGCGACAACCGAGTCTTTGCCAGCTTTCATATCATACTCTGCCAAG     2085
Gohir.D07G208500  TCATATAGGACTAATGCGACAACCGAGTCTTTGCCAGCTTTCATATCATACTCTGCCAAG     2085


CotAD_04861       ------------------------------------------------------------     1845
Gh_D07G2054       AGGGCATCTGAAAGACAAGAACGAGGTTCCCTTGAAGAGAACATTGACATTAGGGAGGCC     2220
GhCLASP2          AGGGCATCTGAAAGACAAGAACGAGGTTCCCTTGAAGAGAACATTGACATTAGGGAGGCC     2145
Gohir.D07G208500  AGGGCATCTGAAAGACAAGAACGAGGTTCCCTTGAAGAGAACATTGACATTAGGGAGGCC     2145
```

```
CotAD_04861       ------------------------------------------------------------     1845
Gh_D07G2054       AGGCGGTCTGTAAATCCACATGTTGACAGGCAATATTTGGACACACCTTATAGAGATGTA     2280
GhCLASP2          AGGCGGTCTGTAAATCCACATGTTGACAGGCAATATTTGGGCACACCTTATAGAGATGTA     2205
Gohir.D07G208500  AGGCGGTCTGTAAATCCACATGTTGACAGGCAATATTTGGACACACCTTATAGAGATGTA     2205


CotAD_04861       ------------------------------------------------------------     1845
Gh_D07G2054       AACTCTCGGGATTTACAGAACAATCATGTTCCAAACTTCCAGAGGCCACTATTGAGAAAG     2340
GhCLASP2          AACTCTCGGGATTTACAGAACAATCATGTTCCAAACTTCCAGAGGCCACTATTGAGAAAG     2265
Gohir.D07G208500  AACTCTCGGGATTTACAGAACAATCATGTTCCAAACTTCCAGAGGCCACTATTGAGAAAG     2265


CotAD_04861       ------------------------------------------------------------     1845
Gh_D07G2054       CATGTAGCTGGGCGGATGTCTGCTGGAAGGAGAAAGAGTTTTGATGATAGCCAGTTGTCA     2400
GhCLASP2          CATGTAGCTGGGCGGATGTCTGCTGGAAGGAGAAAGAGTTTTGATGATAGCCAGTTGTCA     2325
Gohir.D07G208500  CATGTAGCTGGGCGGATGTCTGCTGGAAGGAGAAAGAGTTTTGATGATAGCCAGTTGTCA     2325


CotAD_04861       ---------------------------------------------GATGCCCTAAGCGAG     1860
Gh_D07G2054       CTTGGAGAGATGTCAAGTTATGTTGAAGGTCCAGCTTCTCTTAGTGATGCCCTAAGCGAG     2460
GhCLASP2          CTTGGAGAGATGTCAAGTTATGTTGAAGGTCCAGCTTCTCTTAGTGATGCCCTAAGCGAG     2385
Gohir.D07G208500  CTTGGAGAGATGTCAAGTTATGTTGAAGGTCCAGCTTCTCTTAGTGATGCCCTAAGCGAG     2385
                                                               ***************

CotAD_04861       GGGCTCAGTCCTAGTGCTGATTGGTCTGCCAGGGTTGCTGCTTTTACTTATCTCCGGTCA     1920
Gh_D07G2054       GGGCTCAGTCCTAGTTCTGATTGGTCTGCCAGGGTTGCTGCTTTTACTTATCTCCGGTCA     2520
GhCLASP2          GGGCTCAGTCCTAGTTCTGATTGGTCTGCCAGGGTTGCTGCTTTTACTTATCTCCGGTCG     2445
Gohir.D07G208500  GGGCTCAGTCCTAGTTCTGATTGGTCTGCCAGGGTTGCTGCTTTTACTTATCTCCGGTCA     2445
                  *************** *******************************************

CotAD_04861       TTGTTGCAGCAAGGCCCAAAAGGTATTCAGGAAGTGGTTCAAAACTTCGAGAAGGTAATG     1980
Gh_D07G2054       TTGTTGCAGCAAGGCCCAAAAGGTATTCAGGAAGTGGTTCAAAACTTCGAGAAGGTAATG     2580
GhCLASP2          TTGTTGCAGCAAGGCCCAAAAGGTATTCAGGAAGTGGTTCAAAACTTCGAGAAGGTAATG     2505
Gohir.D07G208500  TTGTTGCAGCAAGGCCCAAAAGGTATTCAGGAAGTGGTTCAAAACTTCGAGAAGGTAATG     2505
                  ************************************************************

CotAD_04861       AAACTGTTTTTCCAGCACTTGGATGATCCCCACCATAAAGTTGCACAGGCTGCCCTTTCA     2040
Gh_D07G2054       AAACTGTTTTTCCAGCACTTGGATGATCCCCACCATAAAGTTGCACAGGCTGCCCTTTCA     2640
GhCLASP2          AAACTGTTTTTCCAGCACTTGGATGATCCCCACCATAAAGTTGCACAGGCTGCCCTTTCA     2565
Gohir.D07G208500  AAACTGTTTTTCCAGCACTTGGATGATCCCCACCATAAAGTTGCACAGGCTGCCCTTTCA     2565
                  ************************************************************
```

```
CotAD_04861       ACTCTTGCAGATATTATTCCATCATGCCGAAAGCCCTTTGAGAGTTACATGGAAAGGATC     2100
Gh_D07G2054       ACCCTTGCTGATATTATTCCATCATGCCGAAAGCCCTTTGAGAGTTACATGGAAAGGATC     2700
GhCLASP2          ACCCTTGCTGATATTATTCCATCATGCCGAAAGCCCTTTGAGAGTTACATGGAAAGGATC     2625
Gohir.D07G208500  ACCCTTGCTGATATTATTCCATCATGCCGAAAGCCCTTTGAGAGTTACATGGAAAGGATC     2625
                  ** ***** ***************************************************

CotAD_04861       TTACCCCATGTTTTCTCACGGTTAATTGATCCAAAGGAGTTGGTTAGGCAGCCTTGCTCA     2160
Gh_D07G2054       CTACCCCATGTTTTCTCACGGTTAATTGATCCAAAGGAGTTGGTTAGGCAGCCTTGCTCA     2760
GhCLASP2          TTACCCCATGTTTTCTCACGGTTAATTGATCCAAAGGAGTTGGTTAGGCAGCCTTGCTCA     2685
Gohir.D07G208500  TTACCCCATGTTTTCTCACGGTTAATTGATCCAAAGGAGTTGGTTAGGCAGCCTTGCTCA     2685
                   ***********************************************************

CotAD_04861       ATGACGTTGGAAATTGTCAGCAAAACCTATAGCATAGATTCCCTGTTACCTGCTTTGCTT     2220
Gh_D07G2054       ATGACGTTGGAAATTGTCAGCAAAACCTATAGCATAGATTCCCTGTTACCTGCTTTGCTT     2820
GhCLASP2          ATGACGTTGGAAATTGTCAGCAAAACCTATAGCATAGATTCCCTGTTACCTGCTTTGCTT     2745
Gohir.D07G208500  ATGACGTTGGAAATTGTCAGCAAAACCTATAGCATAGATTCCCTGTTACCTGCTTTGCTT     2745
                  ************************************************************

CotAD_04861       CGTTCACTTGATGAACAGCGATCACCAAAGGCAAAATTGGCTGTTATCGAGTTCGCTGTT     2280
Gh_D07G2054       CGTTCACTTGATGAACAGCGATCACCAAAGGCAAAATTGGCTGTTATCGAGTTTGCTATT     2880
GhCLASP2          CGTTCACTTGATGAACAGCGATCACCAAAGGCAAAATTGGCTGTTATCGAGTTCGCTGTT     2805
Gohir.D07G208500  CGTTCACTTGATGAACAGCGATCACCAAAGGCAAAATTGGCTGTTATCGAGTTCGCTGTT     2805
                  ***************************************************** *** **

CotAD_04861       ACTTCCTTCAACAAGCATGCTATGAATTCTGAAGGTTCTAGTAATATTGGCATCTTGAAG     2340
Gh_D07G2054       ACTTCCTTCAACAAGCATGCTATGAATTCTGAAGGTTCTAGTAATATTGGCATCTTGAAG     2940
GhCLASP2          ACTTCCTTCAACAAGCATGCTATGAATTCTGAAGGTTCTAGTAATATTGGCATCTTGAAG     2865
Gohir.D07G208500  ACTTCCTTCAACAAGCATGCTATGAATTCTGAAGGTTCTAGTAATATTGGCATCTTGAAG     2865
                  ************************************************************

CotAD_04861       TTATGGCTTGCTAAACTGACACCATTGGTCCATGATAAAAATACTAAGCTTAAGGATGTA     2400
Gh_D07G2054       TTATGGCTTGCTAAACTGACACCATTGGTCCATGATAAAAATACTAAGCTTAAGGATGTA     3000
GhCLASP2          TTATGGCTTGCTAAACTGACACCATTGGTCCATGATAAAAATACTAAGCTTAAGGATGTA     2925
Gohir.D07G208500  TTATGGCTTGCTAAACTGACACCATTGGTCCATGATAAAAATACTAAGCTTAAGGATGTA     2925
                  ************************************************************

CotAD_04861       GCTATCACTTGCATTATATCTGTATACTCGAATTTTGATCCAACTGCTGTTCTGAATTTT     2460
Gh_D07G2054       GCTATCACTTGCATTATATCTGTATACTCGAATTTTGATCCAACTGCTGTTCTGAATTTT     3060
GhCLASP2          GCTATCACTTGCATTATATCTGTATACTCGAATTTTGATCCAACTGCTGTTCTGAATTTT     2985
Gohir.D07G208500  GCTATCACTTGCATTATATCTGTATACTCGAATTTTGATCCAACTGCTGTTCTGAATTTT     2985
                  ************************************************************

CotAD_04861       ATTCTCAGTTTATCAGTTGAAGAGCAAAATTTCCTCAGACGGGCACTCAAACGGTACACT     2520
Gh_D07G2054       ATTCTCAGTTTATCAGTTGAAAAGCAAAATTTCCTTAGACGGGCACTCAAACGGTACACT     3120
```

```
GhCLASP2          ATTCTCAGTTTATCAGTTGAAGAGCAAAATTTCCTCAGACGGGCACTCAAACGGTACACT     3045
Gohir.D07G208500  ATTCTCAGTTTATCAGTTGAAGAGCAAAATTTCCTCAGACGGGCACTCAAACGGTACACT     3045
                  ********************* ************* ************************

CotAD_04861       CCTCGTATTGAGGTGGATCTGATTAACTATTTGCAGAACAAGAAAGAGAGACAGCGTAAA     2580
Gh_D07G2054       CCTCGTATTGAGGTGGATCTGATTAACTATTTGCAGAACAAGAAAGAGAGACAGCGTAAA     3180
GhCLASP2          CCTCGTATTGAGGTGGATCTGATTAACTATTTGCAGAACAAGAAAGAGAGACAGCGTAAA     3105
Gohir.D07G208500  CCTCGTATTGAGGTGGATCTGATTAACTATTTGCAGAACAAGAAAGAGAGACAGCGTAAA     3105
                  ************************************************************

CotAD_04861       TCATCCTATGATCCATCTGATGTTGTTGGAACTTCATCTGAAGAAGGATATATTGGTGTG     2640
Gh_D07G2054       TCATCCTATGATCCATCTGATGTTGTTGGAACTTCATCTGAAGAAGGATATATTGGTGTG     3240
GhCLASP2          TCATCCTATGATCCATCTGATGTTGTTGGAACTTCATCTGAAGAAGGATATATTGGTGTG     3165
Gohir.D07G208500  TCATCCTATGATCCATCTGATGTTGTTGGAACTTCATCTGAAGAAGGATATATTGGTGTG     3165
                  ************************************************************

CotAD_04861       TCCAAGAAGAGTCTTTTACTTGGAAGATATTCTGCTGGTTCCACTGATGGTGATGGTGGT     2700
Gh_D07G2054       TCCAAGAAGAGTCTTTTACTTGGAAGATATTCTGCTGGTTCCACTGATGGTGATGGTGGT     3300
GhCLASP2          TCCAAGAAGAGTCTTTTACTTGGAAGATATTCTGCTGGTTCCACTGATGGTGATGGTGGT     3225
Gohir.D07G208500  TCCAAGAAGAGTCTTTTACTTGGAAGATATTCTGCTGGTTCCACTGATGGTGATGGTGGT     3225
                  ************************************************************

CotAD_04861       AGGAAGTGGGGTTCCACCCTGGAATCAACCCTGATCACTGGAAATATTGGCCTGGCAACA     2760
Gh_D07G2054       AGGAAGTGGGGTTCCACCCTGGAATCAACCCTGATCACTGGAAATATTGGCCTGGCAACA     3360
GhCLASP2          AGGAAGTGGGGGTCCACCCTGGAATCAACCCTGATCACTGGAAATATTGGCCTGGCAACA     3285
Gohir.D07G208500  AGGAAGTGGGGTTCCACCCTGGAATCAACCCTGATCACTGGAAATATTGGCCTGGCAACA     3285
                  *********** ************************************************

CotAD_04861       TCTGATGAAACTCAGGACAACTTATTTCAGAACTTGGAAACTAGTTCAAACACAAATGTT     2820
Gh_D07G2054       TCTGATGAAACTCAGGACAACTTATTTCAGAACTTGGAAACTAGTTCAAACACAAATGTT     3420
GhCLASP2          TCTGATGAAACTCAGGACAACTTATTTCAGAACTTGGAAACTAGTTCAAACACAAATGTT     3345
Gohir.D07G208500  TCTGATGAAACTCAGGACAACTTATTTCAGAACTTGGAAACTAGTTCAAACACAAATGTT     3345
                  ************************************************************

CotAD_04861       TTTCCTTCCAAAACCAAAGAGTCGTCTTATATGGTTAATTCTATTTGTCAGACCTTGGGG     2880
Gh_D07G2054       TTTCCTTCCAAAACCAAAGAGTCGTCTTATATGGTTAATTCTATTTGTCAGACCTTGGGG     3480
GhCLASP2          TTTCCTTCCAAAACCAAAGAGTCGTCTTATATGGTTAATTCTATTTGTCAGACCTTGGGG     3405
Gohir.D07G208500  TTTCCTTCCAAAACCAAAGAGTCGTCTTATATGGTTAATTCTATTTGTCAGACCTTGGGG     3405
                  ************************************************************

CotAD_04861       TCTCAAACTGGCCAGATAGAGAACTTAGAAAGTAGTGTCAACTTGGAAGGTCTATCTACT     2940
Gh_D07G2054       TCTCAAACTGGCCAGATAGAGAACTTAGAAAGTAGTGTCAACTTGGAAGGTCTATCTACT     3540
GhCLASP2          TCTCAAACTGGCCAGATAGAGAACTTAGAAAGTAGTGTCAACTTGGAAGGTCTATCTACT     3465
Gohir.D07G208500  TCTCAAACTGGCCAGATAGAGAACTTAGAAAGTAGTGTCAACTTGGAAGGTCTATCTACT     3465
```

```
                ************************************************************

CotAD_04861      CCACAACTGGAAATTAATGGCCTGAGTAGATTTGACACTTTGGAGACCACTGAAGGAGCT     3000
Gh_D07G2054      CCACAACTGGAAATTAATGGCCTGAGTAGATTTGACACTTTGGAGACCACTGAAGGAGCT     3600
GhCLASP2         CCACAACTGGAAATTAATGGCCTGAGTAGATTTGACACTTTGGAGACCACTGAAGGAGCT     3525
Gohir.D07G208500 CCACAACTGGAAATTAATGGCCTGAGTAGATTTGACACTTTGGAGACCACTGAAGGAGCT     3525
                 ************************************************************

CotAD_04861      ACACATAATGGAACATCATCAGAGTTGGATCTTAATCATCTTAAACCTGCAGCTATAAAG     3060
Gh_D07G2054      ACACATAATGGAACATCATCAGAGTTGGATCTTAATCATCTTAAACCTGCAGCTATAAAG     3660
GhCLASP2         ACACATAATGGAACATCATCAGAGTTGGATCTTAATCATCTTAAACCTGCAGCTATAAAG     3585
Gohir.D07G208500 ACACATAATGGAACATCATCAGAGTTGGATCTTAATCATCTTAAACCTGCAGCTATAAAG     3585
                 ************************************************************

CotAD_04861      GTTAGGTCTATGCCAGACACGGGGCCCAGCATTCCTCAAATTCTTCATGTGATCTGCAAT     3120
Gh_D07G2054      GTTAGGTCTATGCCAGACACGGGGCCTAGCATTCCTCAAATTCTTCATGTGATCTGCAAT     3720
GhCLASP2         GTTAGGTCTATGCCAGACACGGGGCCTAGCATTCCTCAAATTCTTCATGTGATCTGCAAT     3645
Gohir.D07G208500 GTTAGGTCTATGCCAGACACGGGGCCTAGCATTCCTCAAATTCTTCATGTGATCTGCAAT     3645
                 ************************** *********************************

CotAD_04861      GGGAACGATGAGAGCCCTACTGCCAGTAAGCACAATGCACTACAGCAATTACATGAAATT     3180
Gh_D07G2054      GGGAACGATGAGAGCCCTACTGCCAGTAAGCACAATGCACTACAGCAATTACATGAAATT     3780
GhCLASP2         GGGAACGATGAGAGCCCTACTGCCAGTAAGCACAATGCACTACAGCAATTACATGAAATT     3705
Gohir.D07G208500 GGGAACGATGAGAGCCCTACTGCCAGTAAGCACAATGCACTACAGCAATTACATGAAATT     3705
                 ************************************************************

CotAD_04861      TCTGTGGCCAATGATCTCTCTGTTTGGACCAAGTATGCCAATCAGATTTTGACAGCTGTA     3240
Gh_D07G2054      TCTGTGGCCAATGATCTCTCTGTTTGGACCAAGTATGCCAATCAGATTTTGACAGCTGTA     3840
GhCLASP2         TCTGTGGCCAATGATCTCTCTGTTTGGACCAAGTATGCCAATCAGATTTTGACAGCTGTA     3765
Gohir.D07G208500 TCTGTGGCCAATGATCTCTCTGTTTGGACCAAGTATGCCAATCAGATTTTGACAGCTGTA     3765
                 ************************************************************

CotAD_04861      CTTGAGGTTTTAGATGATTCTGATTTCTCAATCAGAGAGCTTGCCCTGTCATTGATAATT     3300
Gh_D07G2054      CTTGAGGTTTTAGATGATTCTGATTTCTCAATCAGAGAGCTTGCCCTGTCATTGATAATT     3900
GhCLASP2         CTTGAGGTTTTAGATGATTCTGATTTCTCAATCAGAGAGCTTGCCCTGTCATTGATAATT     3825
Gohir.D07G208500 CTTGAGGTTTTAGATGATTCTGATTTCTCAATCAGAGAGCTTGCCCTGTCATTGATAATT     3825
                 ************************************************************

CotAD_04861      GAAATGCTTAAAAACCAGGAAGGTGTTATGGGAGATTCTGTTGAAATAGTGATCGAGAAG     3360
Gh_D07G2054      GAAATGCTTAAAAACCAGAAAGGTGTTATGGGAGATTCTGTTGAAATAGTGATCGAGAAG     3960
GhCLASP2         GAAATGCTTAAAAACCAGAAAGGTGTTATGGGAGATTCTGTTGAAATAGTGATCGAGAAG     3885
Gohir.D07G208500 GAAATGCTTAAAAACCAGAAAGGTGTTATGGGAGATTCTGTTGAAATAGTGATCGAGAAG     3885
                 ****************** *****************************************
```

```
CotAD_04861       CTGCTTCATGTAATGAAGGATATTGTTCCAAAAGTTTCAAATGAAGCTGAGCACTGCTTG    3420
Gh_D07G2054       CTGCTTCATGTAATGAAGGATATTGTTCCAAAAGTTTCAAATGAAGCTGAGCACTGCTTG    4020
GhCLASP2          CTGCTTCATGTAATGAAGGATATTGTTCCAAAAGTTTCAAATGAAGCTGAGCACTGCTTG    3945
Gohir.D07G208500 CTGCTTCATGTAATGAAGGATATTGTTCCAAAAGTTTCAAATGAAGCTGAGCACTGCTTG    3945
                  ************************************************************

CotAD_04861       AACACTGTGTTGTCCGAGTATGATCCATTCAGATGTTTAAGTGTTATTGTACCTTTATTA    3480
Gh_D07G2054       AACACTGTGTTGTCCGAGTATGATCCATTCAGATGTTTAAGTGTTATTGTACCTTTATTA    4080
GhCLASP2          AACACTGTGTTGTCCGAGTATGATCCATTCAGATGTTTAAGTGTTATTGTACCTTTATTA    4005
Gohir.D07G208500 AACACTGTGTTGTCCGAGTATGATCCATTCAGATGTTTAAGTGTTATTGTACCTTTATTA    4005
                  ************************************************************

CotAD_04861       GTTACTGAAGATGAGAAGACTTTAGTTATTTGCATCAACTGTTTAACAAAGCTTGTGGGA    3540
Gh_D07G2054       GTTACTGAAGATGAGAAGACTTTAGTTATTTGCATCAACTGTTTAACAAAGCTTGTGGGA    4140
GhCLASP2          GTTACTGAAGATGAGAAGACTTTAGTTATTTGCATCAACTGTTTAACAAAGCTTGTGGGA    4065
Gohir.D07G208500 GTTACTGAAGATGAGAAGACTTTAGTTATTTGCATCAACTGTTTAACAAAGCTTGTGGGA    4065
                  ************************************************************

CotAD_04861       CGGCTATCACAGGAGGAACTAACGGCTCAGTTGCCCTCATTTTTGCCTGCTCTTTTTGAA    3600
Gh_D07G2054       CGGCTATCACAGGAGGAACTAACGGCTCAGTTGCCCTCATTTTTGCCTGCTCTTTTTGAA    4200
GhCLASP2          CGGCTATCACAGGAGGAACTAACGGCTCAGTTGCCCTCATTTTTGCCTGCTCTTTTTGAA    4125
Gohir.D07G208500 CGGCTATCACAGGAGGAACTAACGGCTCAGTTGCCCTCATTTTTGCCTGCTCTTTTTGAA    4125
                  ************************************************************

CotAD_04861       GCTTTCGGAAACCAGAGTGCTGATGTCCGTAAGACTGTCGTTTTTTGTTTGGTGGATATT    3660
Gh_D07G2054       GCTTTCGGAAACCAGAGTGCTGATGTCCGTAAGACTGTCGTTTTTTGTTTGGTGGATATT    4260
GhCLASP2          GCTTTCGGAAACCAGAGTGCTGATGTCCGTAAGACTGTCGTTTTTTGTTTGGTGGATATT    4185
Gohir.D07G208500 GCTTTCGGAAACCAGAGTGCTGATGTCCGTAAGACTGTCGTTTTTTGTTTGGTGGATATT    4185
                  ************************************************************

CotAD_04861       TACATAATGCTTGGGAAATCATTTTTGCCTCACTTGGAGGGGCTTAACAGCACACAATTG    3720
Gh_D07G2054       TACATAATGCTTGGGAAATCATTTTTGCCTCACTTGGAGGGGCTTAACAGCACACAATTG    4320
GhCLASP2          TACATAATGCTTGGGAAATCATTTTTGCCTCACTTGGAGGGGCTTAACAGCACACAATTG    4245
Gohir.D07G208500 TACATAATGCTTGGGAAATCATTTTTGCCTCACTTGGAGGGGCTTAACAGCACACAATTG    4245
                  ************************************************************

CotAD_04861       CGGCTGGTTACAATTTATGCCAATCGGATATCACAGGCCAGGACCGGCACACCTATAGAT    3780
Gh_D07G2054       CGGCTGGTTACAATTTATGCCAATCGGATATCACAGGCCAGGACCGGCACACCTATAGAT    4380
GhCLASP2          CGGCTGGTTACAATTTATGCCAATCGGATATCACAGGCCAGGACCGGCACACCTATAGAT    4305
Gohir.D07G208500 CGGCTGGTTACAATTTATGCCAATCGGATATCACAGGCCAGGACCGGCACACCTATAGAT    4305
                  ************************************************************

CotAD_04861       GCTGGTCATGAATAG                                                 3795
Gh_D07G2054       GCTGGTCATGAATAG                                                 4395
```

```
GhCLASP2          GCTGGTCATGAATAG                                                                4320
Gohir.D07G208500 GCTGGTCATGAATAG                                                                 4320
                  ***************
```

Supplementary Figure 2 Multiple sequence alignment of *GhCLASP2*, *Gh_D07G2054*, *CotAD_04861* and *Gohir.D07G208500*. The black underlined letters indicates the nucleotide sequence of *GhCLASP2* gene for the *GhCLASP2*-RNAi vector construction.

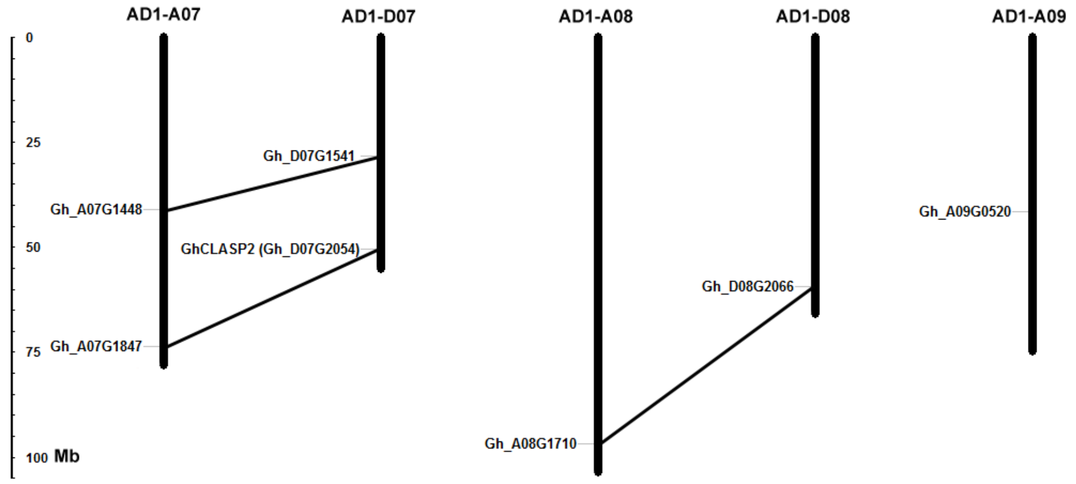

Supplemental Figure 3 Physical map of *CLASP* family genes in cotton. *Gh_A07G1448*, *Gh_D07G1541*, *Gh_A09G0520*, *Gh_D08G2066*, *Gh_A08G1710*, *Gh_A07G1847* and *GhCLASP2*(*Gh_D07G2054)* from *G. hirsutum* acc.TM-1. The scale represents megabases (Mb).

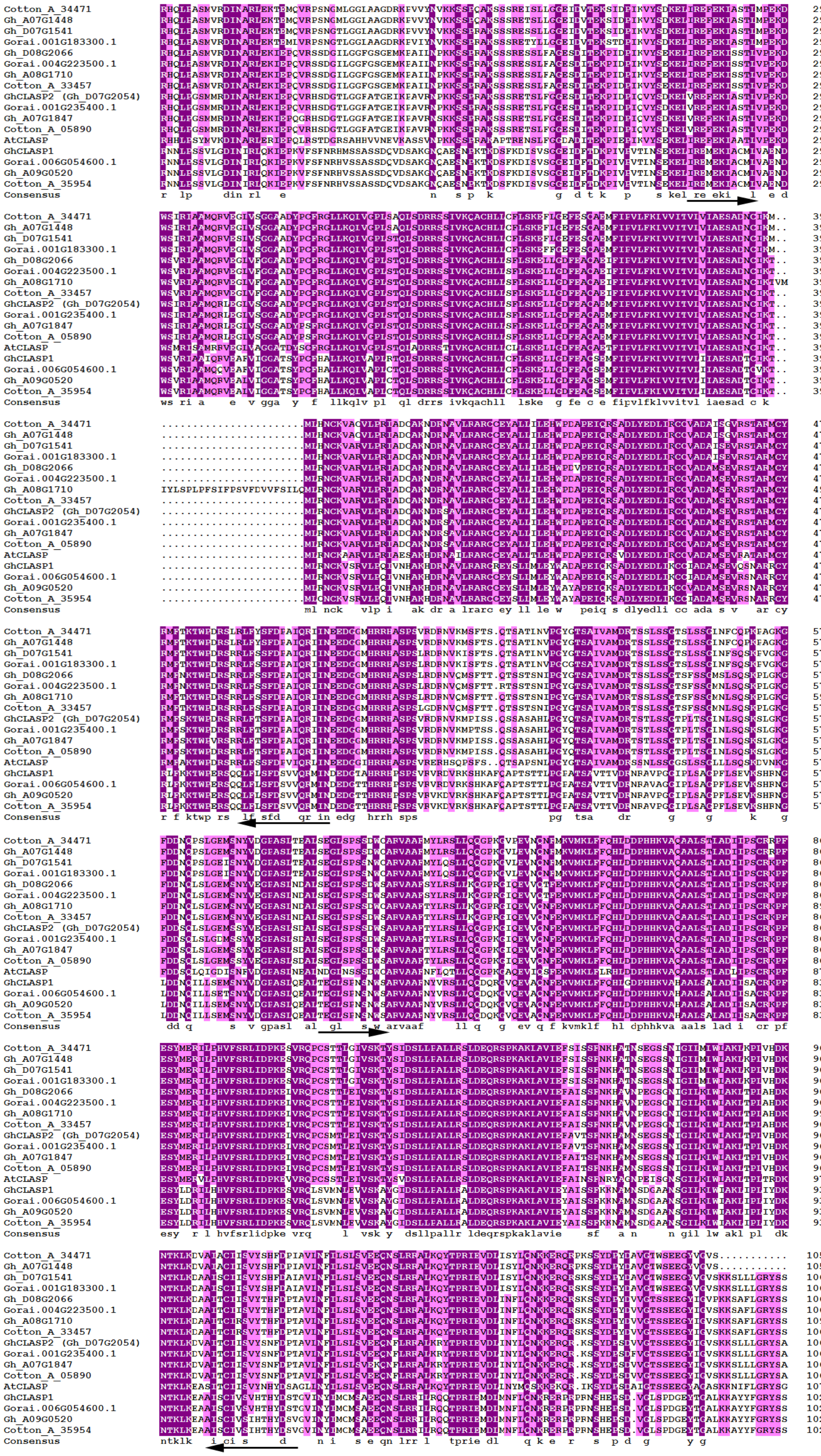

Supplementary Figure 4 Multiple alignments of the deduced amino acid sequences of *CLASP* family genes proteins (Cotton_A_35954, Cotton_A_34471, Cotton_A_33457 and Cotton_A_05890 from *G. arboreum*, Gorai.001G183300.1, Gorai.001G235400.1, Gorai.006G054600.1 and Gorai.004G223500.1 from *G. raimondii*, Gh_A07G1448 Gh_D07G1541, Gh_A09G0520, Gh_D08G2066, Gh_A08G1710 and Gh_A07G1847 from *G. hirsutum* acc.TM-1, GhCLASP2 (Gh_D07G2054) and GhCLASP1 from *G. hirsutum* acc.Xinluzao33) and AtCLASP proteins. The black arrows marked with two CLASP-N terminal domain of protein.

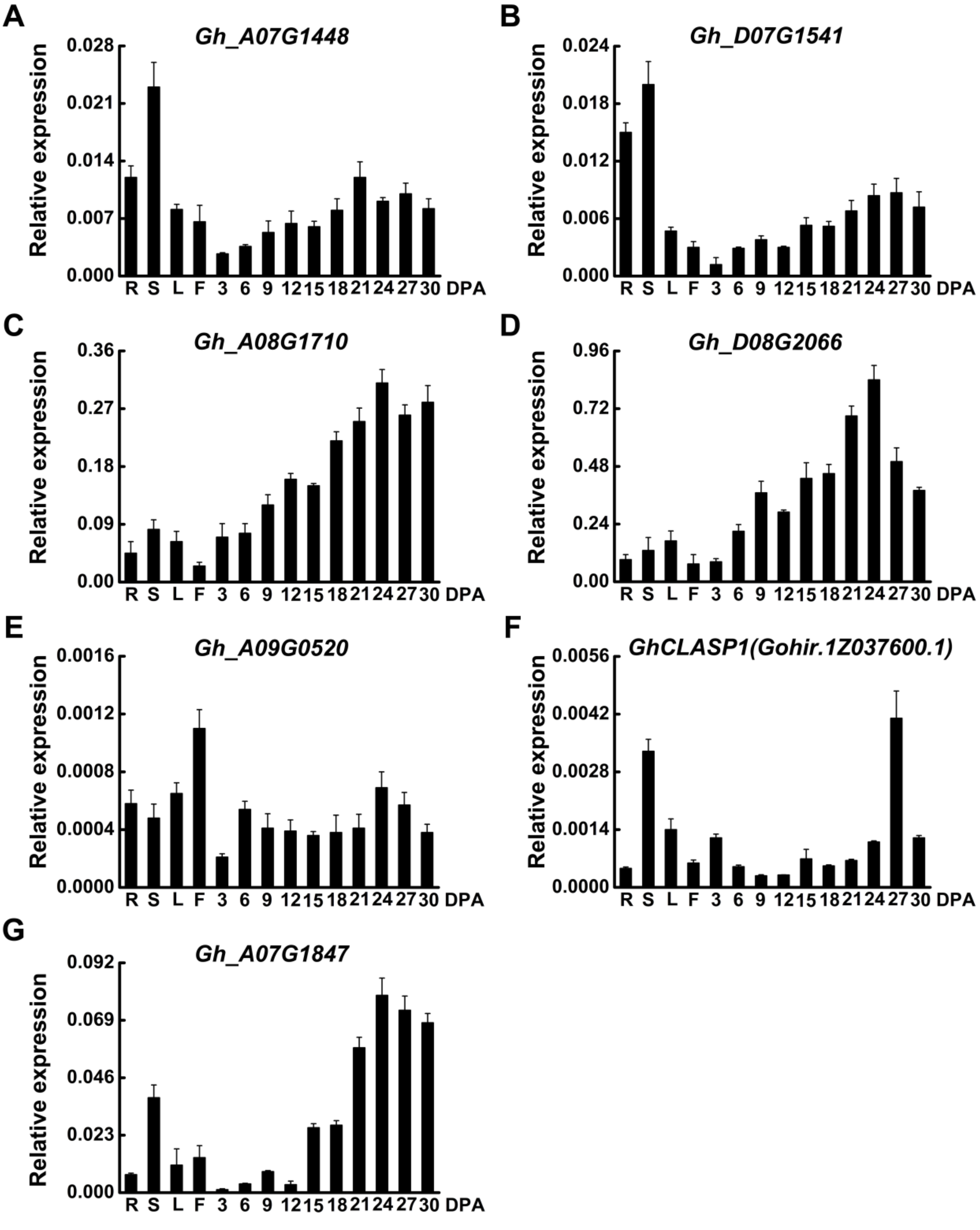

Supplementary Figure 5 qRT-PCR analysis of seven *GhCLASP2* homologs expression in various cotton tissues. R, root; S, stem; L, leaf; F, flower (0 DPA), and

fibers of 3, 6, 9, 12, 15, 18, 21, 24, 27 and 30 DPA. Data are means ±SD. Error bars represent the standard deviation of triplicate experiments, and *GhUBQ7* was used as an internal control.

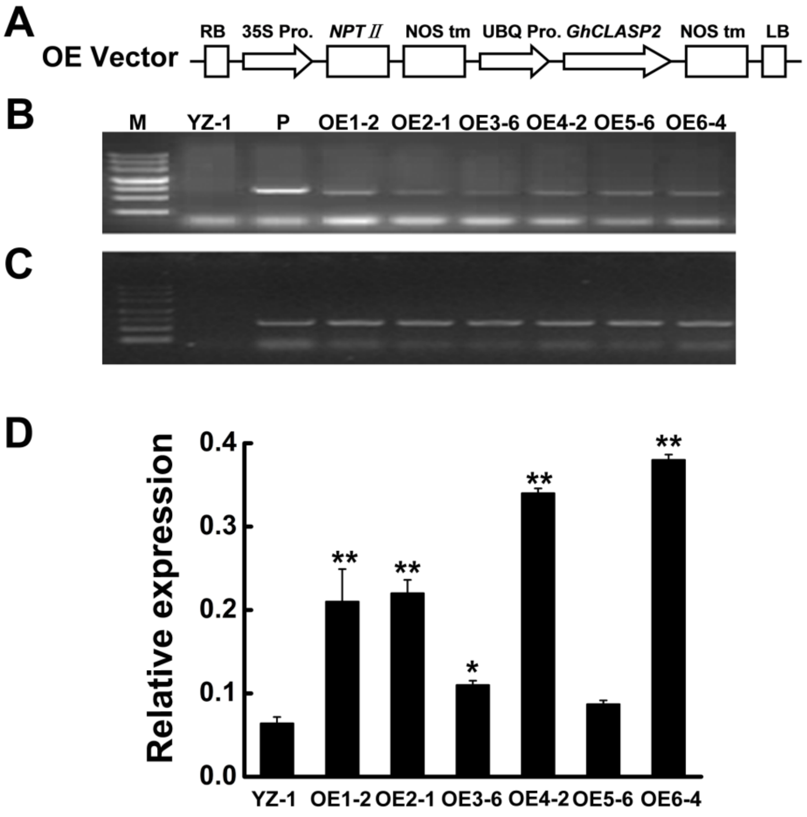

Supplementary Figure 6 Identification and expression analysis of *GhCLASP2*-overexpressed transgenic cotton lines. (**A**)Schematic representation of constructions of overexpression vector. 35S pro., CaMV 35S promoter; UBQ pro., UBiquitin promoter; NOS tm, NOS terminor; *NPT II*, neomycin phosphotransferase II gene; *GhCLASP2*, cDNA sequence of the *GhCLASP2* gene; RB, right border; LB, left border; OE Vector, overexpression of *GhCLASP2* vector. (**B**) PCR analysis of transgenic plants to determine the NPTII (700 bp). (**C**) PCR analysis of transgenic plants to determine the Vectors and gene fragments (701 bp). M, molecular weight marker; P, positive control; YZ-1, non-transformed plant; OE1-2, OE2-1, OE3-6, OE4-2, OE5-6 and OE6-4, *GhCLASP2*-overexpressed transgenic plants. (**D**) qRT-PCR analysis of YZ-1 and overexpressed-*GhCLASP2* transgenic cotton lines (OE1-2, OE2-1, OE3-6, OE4-2, OE5-6 and OE6-4). Data are means ±SD. Error bars represent the standard deviation of triplicate experiments, and *GhUBQ7* was used as an internal control in qRT-PCR. P values were determined based on Student's *t* tests. * and ** indicate significant differences at $p < 0.05$ and $p < 0.01$, respectively.

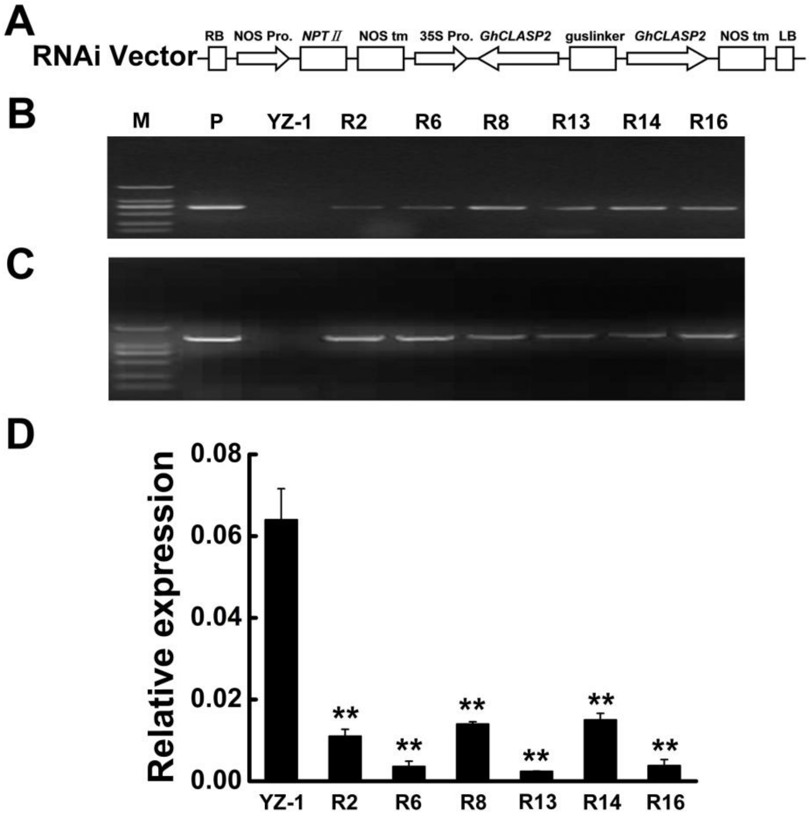

Supplementary Figure 7 Identification and expression analysis of *GhCLASP2*-RNAi transgenic cotton lines. (**A**) Schematic representation of constructions of RNAi vector of *GhCLASP2*. 35S pro., CaMV 35S promoter; NOS tm, NOS terminor; *NPT II*, neomycin phosphotransferase II gene; *GhCLASP2*, the specific interference target cDNA sequence of the *GhCLASP2* gene; RB, right border; LB, left border; RNAi Vector, RNA interference expression of *GhCLASP2* vector. (**B**) PCR analysis of transgenic plants to determine the NPTII (700 bp). (**C**) PCR analysis of transgenic plants to determine the Vectors and gene fragments (1174 bp). M, molecular weight marker; P, positive control; YZ-1, non-transformed plant; R2, R6, R8, R13, R14 and R16, the *GhCLASP2*-RNAi transgenic plants. (**D**) qRT-PCR analysis of YZ-1 and *GhCLASP2*-RNAi transgenic cotton lines (R2, R6, R8, R13, R14 and R16). Data are means ±SD. Error bars represent the standard deviation of triplicate experiments, and *GhUBQ7* was used as an internal control in qRT-PCR. P values were determined based on Student's *t* tests. * and ** indicate significant differences at $p < 0.05$ and $p < 0.01$, respectively.

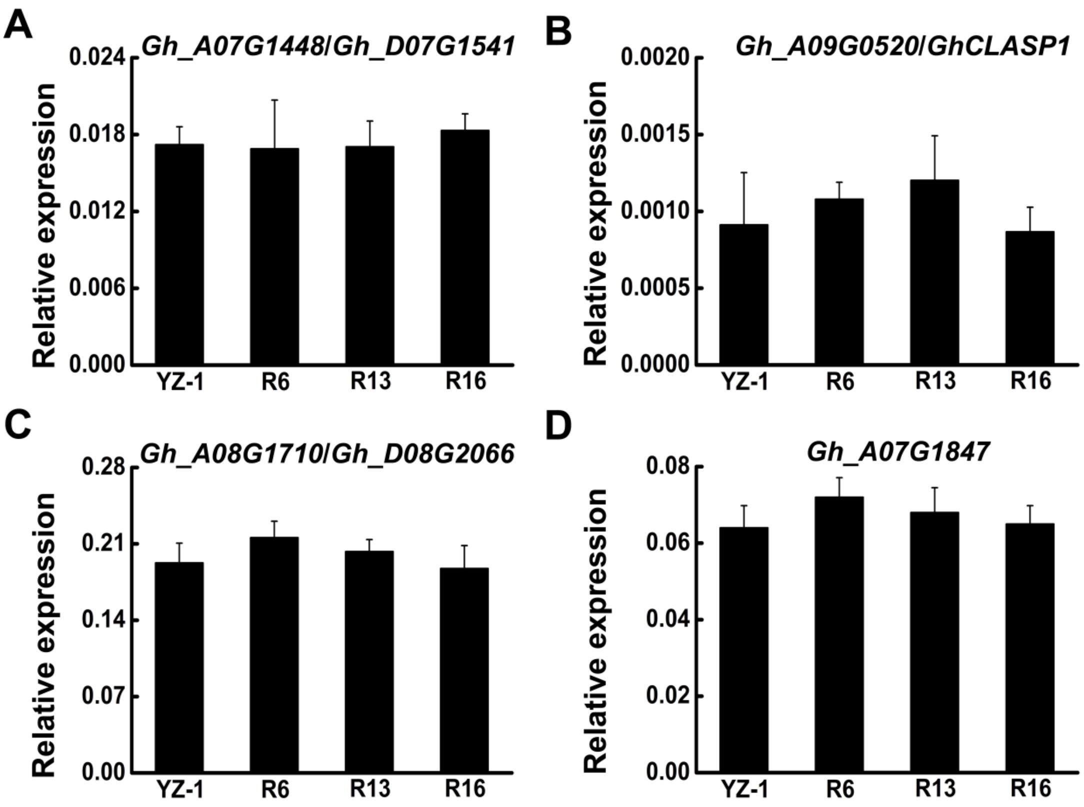

Supplementary Figure 8 Transcript levels of *Gh_A07G1448*/*Gh_D07G1541*, *Gh_A08G1710*/*Gh_D08G2066*, *Gh_A09G0520*/*GhCLASP1* and *Gh_A07G1847* of *GhCLASP2*-RNAi transgenic plant lines (R6, R13 and R16) and the WT (YZ-1). Data are means ±SD. Error bars represent the standard deviation of triplicate experiments, and *UBQ7* was used as an internal control in qRT-PCR.
